# Can different osteotomies have an influence on surgically assisted rapid maxillary expansion? A systematic review

**DOI:** 10.1186/s13005-024-00415-3

**Published:** 2024-03-08

**Authors:** Selene Barone, Francesco Bennardo, Marianna Salviati, Elena Calabria, Tecla Bocchino, Ambra Michelotti, Amerigo Giudice

**Affiliations:** 1 School of Dentistry, Department of Health Sciences, Magna Graecia University of Catanzaro, Viale Europa, Catanzaro, 88100 Italy; 2 Department of Neurosciences, Reproductive Sciences and Oral Sciences, University of Naples Federico II, Naples, Italy

**Keywords:** Maxillary expansion, Surgically assisted rapid maxillary expansion, Pterygomaxillary disjunction, Segmental Le Fort I osteotomy

## Abstract

The purpose of this study was to systematically review the randomized and non-randomized clinical trials (RCT; nRCT) concerning the different available osteotomies for surgically assisted rapid maxillary expansion (SARME): pterygomaxillary disjunction (SARME + PD vs SARME-PD) and segmental Le Fort I osteotomy (2-piece vs 3-piece). Outcomes focused on skeletal, dental, upper airway changes, complications, and relapse. Two authors investigated five databases (PubMed, Cochrane Library, Google Scholar, Scopus, Web of Science) until August 2023. The Cochrane Collaboration Tool and the Newcastle–Ottawa scale were used for the quality assessment of the included RCTs and nRCTs, respectively. A total of 554 articles were retrieved and after duplicates removing and full-text reading, 40 studies were included. Two RCTs showed a low risk of bias, one an unclear risk and one a high risk. Among the non-RCTs, 15 studies showed a good quality, while 21 exhibited a fair quality score. SARME + PD resulted in more homogeneous posterior bone expansion, with minimal dental effects. No difference between 2-piece and 3-piece in asymmetric expansion was observed, although 3SO showed 1–2 mm of more transverse increase. The oropharynx minimum cross-sectional area, the nasopharynx and the oropharynx volume were greater in SARME + PD. Both dental and bone relapse can occur but no differences between the groups were observed. All osteotomies guaranteed a correction of transverse maxillary deficiency. Lower side effects were described in SARME + PD. Two-piece and 3-piece segmental Le Fort I osteotomies did not show any differences in the symmetry and amount of expansion.

**Supplementary Information:**

The online version contains supplementary material available at 10.1186/s13005-024-00415-3.

## Introduction

Transversal maxillary discrepancies represent a common type of malocclusion among young and adult people with an incidence range of 8–18% of patients requiring orthodontic evaluation [1]. The resulting crossbite can be distinguished according to the causal factor: in most cases a maxillary constriction is outlined, at other times a maxillary constriction is associated with an increased mandibular width but sometimes a normal maxillary transverse dimension is associated with an increased mandibular width [2]. A multifactorial etiology can be recognized for transversal maxillary deficit, including congenital, developmental (thumb sucking, breathing alterations), iatrogenic (cleft palate repair), or traumatic factors [3, 4]. When a diagnosis of maxillary constriction occurred, it’s useful to improve the transversal discrepancy, to increase the arch perimeter for a correct dental alignment and arch coordination, to enlarge the palatal vault providing an adequate space for the tongue, and to increase the nasal cavity width for the improvement of nasal breathing [5, 6].

In patients with skeletal maturity, surgically assisted rapid maxillary expansion (SARME) is indicated for the treatment of extreme transverse maxillary hypoplasia [2, 7]. Orthodontic therapy can camouflage discrepancies less than 5 mm (mm) with orthopedic forces alone, but when a higher transversal maxillary discrepancy occur, SARME is considered the gold standard [8].

To date, there is no consensus in the literature for the best osteotomies to be performed in SARME. All maxillary joints and suture lines have been found to contribute differently to resistance to maxillary expansion [3, 5, 9–19]. The areas of resistance have been classified as anterior support (piriform aperture pillars), lateral support (zygomatic buttresses), posterior support (pterygoid junctions), and median support (midpalatal synostosed suture) [2, 20]. As clinical consequence, the surgical procedure can include Le Fort I osteotomy in association with other specific osteotomies: 1) pterygomaxillary disjunction (PD); 2) median palatal suture osteotomy that can be defined as segmental 2-piece osteotomy (2S); 3) two paramedian osteotomies between the lateral incisor and the canine (3-piece; 3S). In order to choose the best treatment, to guarantee the best predictable and stable results, to achieve the minimal patient morbidity, and to avoid complications and relapse at the long-term, more details on maxillary osteotomies for SARME are needed [1, 5, 12, 16, 21–23]. It is essential to focus on which osteotomies could decrease any resistance to maxillary expansion, achieving an adequate transversal correction without periodontal damage [5].

The purpose of this study was to systematically review the available randomized and non-randomized clinical trials to analyze the different osteotomies used in SARME in terms of skeletal, dentoalveolar, upper airways outcomes, relapse, and complications.

## Materials and methods

A systematic review was conducted following PRISMA guidelines and PICOS (participants, intervention, comparisons, outcomes, and study design) criteria: (P) Human participants of any age, sex, and ethnicity, (I) diagnosed with transverse maxillary deficiency equal to or greater than 5 mm, exhibiting a mono- or bilateral crossbite, (C) included in randomized- and non-randomized- clinical trials (O) that evaluated the various osteotomies employed in SARME in terms of skeletal, dentoalveolar, upper airway outcomes, relapse, and complications.

### Search strategy and study selection

The PubMed, Cochrane Library, Google Scholar, Scopus, Web of Science databases were investigated up to August 2023 for the electronic search. The term sequence used in PubMed search was: “((maxillary expansion[MeSH Terms]) AND (osteotomy, le fort[MeSH Terms])) AND (orthognathic surgery[MeSH Terms])”, “(((maxillary expansion[MeSH Terms]) AND (orthognathic surgery[MeSH Terms])) AND (segmental osteotomy)”, “(((maxillary expansion[MeSH Terms]) AND (orthognathic surgery[MeSH Terms])) AND (pterygomaxillary disjunction)) OR (segmental le fort I osteotomy)”, “((orthognathic surgery[MeSH Terms]) AND (le fort osteotomy[MeSH Terms])) AND (2-piece or 3 piece)”. The term sequences used in the other search databases were: “[surgically assisted rapid maxillary expansion] AND [pterygomaxillary disjunction] OR [segmental le fort I osteotomy]”, “((“maxillary expansion”) AND (segmental AND le AND fort AND I AND osteotomy) OR (pterygomaxillary AND disjunction))”, “(“maxillary expansion”) AND (segmental le fort I osteotomy) OR (pterygomaxillary disjunction)”. To complete the search strategy, a manual search was performed, considering the reference lists of the included studies. No restriction of language or publication date was applied.

**Fig. 1:**
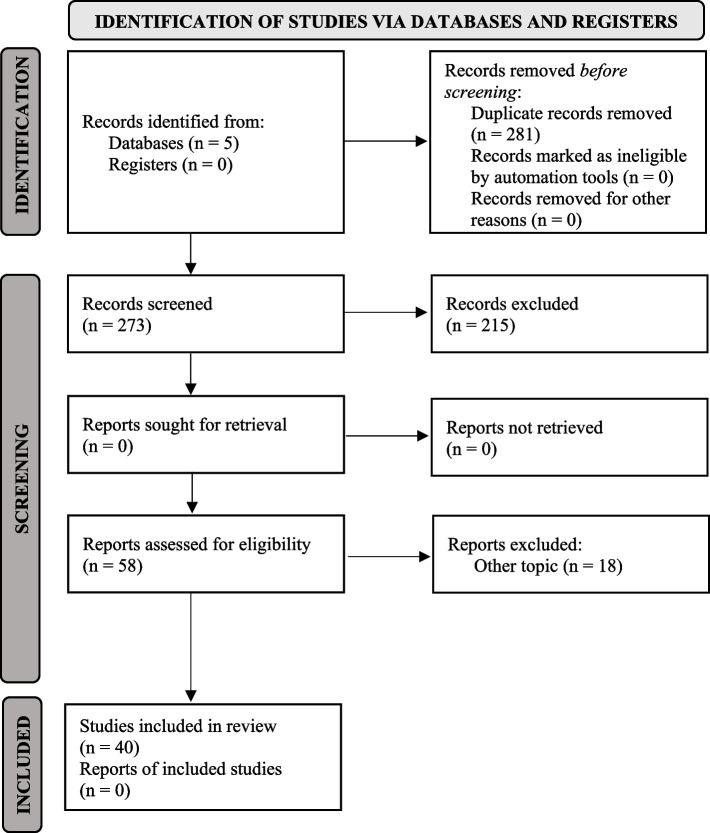
PRISMA flow diagram of the selection process

The electronic search was conducted independently by two investigators (SB and MS). The screening of titles and abstracts allowed to assess the randomized- and non-randomized- clinical trials (RCT; nRCT) for eligibility (Fig. 1). Full-text reading was scheduled when missing information persisted. Any disagreement between the two authors were discussed with an expert supervisor (AG). The inter-rater reliability between the two investigators (SB and MS) was determined calculating the Cohen’s kappa coefficient (k).

### Data extraction process

Data extraction was separately performed by the same two authors (SB and MS) from eligible studies. The recorded data were the following: author, publication date, country of the included studies, number of the included patients, type of intervention, methodological procedure, outcomes, studies’ results, and author’s conclusion.

### Assessment of methodological quality

The analysis of the methodological quality was independently conducted by the two investigators (SB and MS). Each included RCT was evaluated using the Cochrane Collaboration Tool [24]. The Cochrane Collaboration Tool is a validated instrument, assessing the study design and methodology according to six domains (selection bias, performance bias, detection bias, attrition bias, reporting bias, other bias). Each domain expresses the risk of bias in the format “low”,” “high”, or “unclear”. Non-randomized clinical trials were assessed using the Newcastle–Ottawa Quality Rating Scale. Each study can show poor, fair, or good/high quality based on a star rating system with eight elements in three domains (selection, comparability, and outcome) [25].

## Results

### Study selection

maxillary expansion had no significant difference between A total of 554 articles were retrieved from five databases. The duplicates were then removed, leaving a total of 273 articles. The articles were then screened on the basis of title and abstract and 58 articles were selected (Suppl. Tab. 1). After a full-text analysis, 18 articles were dismissed because they related to other topics. A total of forty 40 articles were finally included in the systematic review (Table 1). The inter-rater agreement coefficient was *k* = 0.91.

**Table 1:** List of the included studies

Authors; Year; Journal;	Study design	Patients; Age; Transversal maxillary deficit (TMD)	Surgical approach: osteotomy	Radiographic exam; Timing of evaluation	Outcomes variables	Results and conclusion	Methodological quality
Matteini and Monnaerts; 2001 AJODO [26]	Prospective study	20 patients Mean age: 20 years NA	Le Fort I osteotomy + 2-piece osteotomy + Pterygomaxillary disjunction (PD)	Clinical records and plaster casts - before surgery -2–3 weeks later	The most palatal points at the gingival margin of the first molars, the first premolars, and the canines; The contact points on the mesial surface of the first molars; The mesial surface of the first premolar; The distal surface of the central incisors; The most facial point of the most prominent central incisor.	Expansion averaged 29.9% at the level of the canines, 28.3% at the level of the first premolars, and 20.8% (SD 7.2) at the level of the first molars. The expansion percentages must be related to the original widths at the different levels to learn the differential movement of the anterior and posterior parts of the segments. The average original intercanine and interpremolar distances were 75% and 77.7%, respectively, of the average original intermolar distance.	Fair
Babacan et al. 2006 Angle Orthod [27]		10 subjects mean age: 18.70 ± 2.54 years	horizontal osteotomy + pterygomaxillary disjunction, + midpalatal suture separation	Acoustic rhinometry; occlusal radiograph Mean follow-up: 6.19 ± 0.16	nasal volume (in cc) at the beginning of treatment (T1) and at the end of the retention period (T2); Study casts at T1 and T2 to analyze changes in intermolar and intercanine width	All the subjects demonstrated sutural opening. The mean intercanine expansion was 6.50 ± 1.97 mm for SARME. The mean intermolar expansion was 8.50 ± 3.82 mm for SARME. A significant volume increase was observed between the T1 and T2 measurements.	Good
Goldenberg et al.; 2007 J Craniofacial Surgery [28]	Prospective study	15 patients; mean age: 24.47 ± 5.79; TMD > 5 mm, a maxilla-mandibular transverse differential index > 5 mm, and a crossbite	Le Fort I osteotomy + 2-piece osteotomy - Pterygomaxillary disjunction (PD)	CT before surgery after surgery (6 months)	- Width of the nasal floor, - Distance between the palatine process of the maxilla, - Distance between the inferior margin of the maxillary alveolar process - The distance between the greater palatine foramina - The angle between the anterior nasal spine and the inferior margin of the alveolar bone - The angle formed by anterior nasal spine and greater palatine foramina,	Mean appliance expansion was 11.47 + 1.65 mm. The cross-sectional area of the maxilla was significantly increased. The angle between the anterior nasal spine and the inferior margin of the alveolar bone increased in a significant manner (P G 0.05). A statistically significant increase in maxillary width was also observed in the intermediate portion of the maxilla. Measurements of the distance between the right and left greater palatine foramina, and the angle between the anterior nasal spine and the greater palatine foramina did not show differences that were statistically significant. No complications; All patients spontaneously reported a clinically significant improvement in nasal airflow and breathing.	Fair
Landes et al.; 2009 JOMS [29]	Cohort study	50 patients bipartite osteotomy (*n* = 28) tripartite osteotomy (*n* = 22) TMD	Le Fort I osteotomy + 2-piece or 3-piece osteotomy + Pterygomaxillary disjunction (PD)	CT - before surgery - after surgery	- Transverse skeletal and dental expansion, - Segmental skeletal inclination, - Dental tipping - Bone resorption - Tooth inclination	Skeletal transverse widening: The biggest decrease in skeletal transverse widening from anterior to posterior was found in the tripartite, bone-borne, age older than 20 years, pterygoid osteotomy group. Dental transverse widening: The greatest decline in dental transverse widening from anterior to posterior was found in the tripartite, bone-borne, age younger than 20 years, pterygoid osteotomy group. Dental tipping: Outward rotation was seen in the tripartite, age older than 20 years, pterygoid osteotomy group, irrespective of the distractor used. Inward rotation was seen in the bipartite, bone borne, age older than 20 years, no pterygoid osteotomy group. Bone resorption: The greatest loss in vestibular bone substance was detected in the bipartite, bone-borne, age younger than 20 years, no pterygoid osteotomy group in the molars and in the tripartite, age older than 20 years group in the premolars. The palatal bone substance increased in the tripartite, tooth-borne group in the molars and in the tripartite, pterygoid osteotomy group in the premolars.	Good
Marchetti et al.; 2009 J Cranio Maxillofac Surg [30]	Retrospective study	20 patients: SARME (*n* = 10) Le Fort I bipartition (*n* = 10) SARME: mean age of 23.5 years; Le Fort I bipartition: mean age of 27.75 years SARME: TMD > 6 mm; Le Fort I bipartition: TMD < 6 mm	Le Fort I osteotomy + 2-piece osteotomy + Pterygomaxillary disjunction (PD) ± Down fracture	- Plaster models of the maxilla - pre-expansion (T1), - post-expansion (T2), - 2 years post-expansion (T3)	- Intercanine distance (from the cusp of the 1.3 tooth to the cusp of the 2.3 tooth) - intermolar (from the 1.6 tooth mesio-palatine cusp to the 2.6 tooth mesio-palatine cusp)	In the SARPE group, the increase in the intercanine distance between T1 and T2 ranged from 4.5 to 10.5 mm, whereas the change from T2 to T3, or the relapse, ranged from + 1 to -6 mm. Similarly, the intermolar distance increased by 5–9.5 mm between T1 and T2, whereas the relapse ranged from + 3 to -3.5 mm In the Le Fort I bipartition group, the increase in the intercanine distance ranged from 0 to 4.5 mm, which was smaller than that in the SARPE group, whereas the relapse ranged from + 3.5 to -2.5 mm. In contrast, the intermolar distance was increased by 2.5–7.5 mm, and the relapse ranged from 0 to -5 mm. The differences throughout T1-T3 for both treatments were significant (*p* < 0.001). The relapse in the intercanine and intermolar distances between T3 and T2 was smaller for Le Fort I bipartition than for SARPE.	Good
Laudemann et al.; 2009 Oral Maxillofac Surg [31]	Cohort study	50 patients; retrospectively (*n* = 38) prospectively (*n* = 12) SARME + PD (21 patients) SARME -PD (29 patients) Age: 13–50 years TMD	Le Fort I osteotomy + 2-piece or 3-piece osteotomy ± Pterygomaxillary disjunction (PD)	CT - before surgery - after surgery - Mean follow-up: 15 months	- Skeletal internal and external maxillary widths; - Dental width: the most prominent area of the buccal aspect of the posterior teeth (DA.E) and, on the other hand, at the level of the buccal cusp tips (DA.E); - width between the teeth apices, the palatal root; - The dental arch internal width measured at - the most prominent area of the palatal aspect of the teeth (DA.I) as well as at the level of the lingual cusp tips (DA.I); - distance between the buccal tooth apex down to the BAC was measured bilaterally (TA.BAC), then the distance between the buccal tooth apex and the external rim of the buccal alveolar bone (BTA.E). - The distance between the lingual (palatal) tooth apex and the hard palate.	Bigger transverse widening tended to occur more anteriorly directed (first premolar area) in the + PP group and more posteriorly directed (first molar area) in the − PP group. In SARME − PP group there was more parallel distraction from molar level to pterygoid level increase in pterygoid widening and bigger lateral pterygoid bending. For the + PP with BB devices in patients < 20 years, the biggest decline in transverse width along the dental arch was found. For the + PP in BB devices, there was the biggest outward segmental inclination along the dental arch. Biggest bone resorption at premolar level was found in the − PP with BB devices and biggest increase in vestibular bone plate thickness in the premolars (0.29 mm) in the + PP with BB devices.	Good
Seeberger, R. 2010 Journal of Cranio-Maxillo-Facial Surgery [32]	Retrospective clinical mono- centre study	13 patients mean age: 31.23^6.11 TMD ≧ 5 mm	Le Fort I osteotomy + 2-piece osteotomy ± Pterygomaxillary disjunction	Acoustic rhinometry - One month before surgery - 63 months after the operation	Volume of nasal airways divided in the different segments: - Anterior segment of the nasal cavity, - medium segment of the nasal cavity, - posterior segment of the nasal cavity - nasal isthmus	All patients showed a significant enlargement of the nasal volume as result of the palatal transverse distraction. The volumes of the different segments indicate a V-shaped movement of the segments. The gain was greater in the anterior than in the medium and posterior segments. No relapse of the maxillary expansion was observed. Stable orthognathic occlusions were observed 63 months after treatment. All patients reported substantial improvement of nasal respiration.	Good
Hernandez-Alfaro et al.; 2010 JOMS [33]	Retrospective case series	283 patients; Mean age: 18.3 years Severe TMD	Minimally invasive SARPE with total liberation of the anterior, lateral, posterior, and medial buttresses	Clinical evaluation 1-year follow-up	-Amount of dental expansion; - Complications and relapse;	Mean expansion was 9.2 mm (range, 3.0 to 15 mm) at the level of the mesiovestibular cuspid of the first molar expansion. Reintervention was necessary in 3 cases because of the lack of expansion on 1 side. At the 1-year follow-up visit, mean expansion at the canines was 8.0 (range, 3.0 to 13.0), and at the mesiovestibular cuspid of the first molar, it was 8.9 (range, 2.0 to 14.2).	Good
De Assis et al. 2010 Oral and Maxillofacial surgery [34]	Prospective study	13 patients; Mean age of 26.5 years old (from 18 to 38 years old) TMD > 5 mm	subtotal Le Fort I osteotomy, without nasal septum osteotomy, and maxillary disjunction	Clinical follow-up using digital paquimeter: - 2 months post-surgery - 6 months post-surgery - 24 months post-surgery - 36 months post-surgery	Measurement of the alar base by using a digital paquimeter, whose points were laid in the lateral face of the alar insertions	No complications; There was a discrete increase in the 2-month post-operative control period. There was a statistical relevance (*P* < 0.05) in the crossing of the pre-operative period with the 6-, 24-, and 36-month post-operative periods. There were no statistical significant results when the post-operative periods were compared among each other.	Good
Landim et al.; 2011 *Int. J. Med. Sci* [35]	Prospective cohort study	15 patients; Mean age 23.8 years; TMD	Le Fort I osteotomy + 2-piece osteotomy + Pterygomaxillary disjunction (PD)	The frontal cephalometric radiograph - before treatment (T0); - during locking of expander 14 to 18 days following surgery (T1); - at least six months following the locking of the expander (T2)	- Nose width - Position of the nasal septum in relation to the left and right lateral walls of the pyriform aperture	No statistically differences were detected in these variables between sides or between evaluation periods (p > 0.05).	Fair
Laudemann et al.; 2011; Journal of Oral Maxillofacial Surgery [36]	**RCT**	45 patients; 13–50 years Transversal maxillary deficiency	Le Fort I osteotomy + (with) 2-piece or 3-piece osteotomy + (with) /- (without) Pterygomaxillary disjunction (PD)	CT; T0: preoperatively T1: 2.87 ± 1.59 months after expansion	-Maxillary width; -Buccal bone loss; -Transverse width at pterygoid process; - Bending of the pterygoid process.	A lower transverse widening and an increased buccal bone plate were recorded in SARME + PD, in patients younger than 20 years with bone-borne devices. The greatest increase in transverse widening was found in patients with 3-piece osteotomy and tooth-borne devices. The greatest pterygoid lateral bending was found in patients with 2-piece osteotomy; the greatest pterygoid medial bending was found in patients with 3-piece osteotomy.	Good
Landes et al.; 2012 Oral Surg Oral Med Oral Pathol Oral Radiol [37]	Cohort study	77 patients: 2-piece (*n* = 22) 3-piece (*n* = 55); Mean age of 24.3 ± 8.7 years; TMD	Le Fort I osteotomy + 2-piece or 3-piece osteotomy + Pterygomaxillary disjunction (PD)	CT - before surgery - after surgery Mean follow-up: 20.5 months post-operatively	For transverse skeletal maxillary widening: - the distance between the right and left vestibular gingival margins. - the distance between the right and left palatal gingival margins For transverse dental maxillary widening: - the distance between the right and left vestibular cusp tips - the distance between the right and left palatal cusp tips. - vestibular attachment loss in clinical crown length on the central incisors, lateral incisors, canines, and first and second premolars. - angulation of the central and lateral incisors and canines	3SO as well as 2SO increased transverse skeletal width along the dental arch; however, 3SO expanded more symmetrically and led to more general dental tipping. More increase in clinical crown length occurred in 2SO, however, except for the lateral incisors and canines at the osteotomy sites of 3SO. 3SO provoked more inward angulation in the central incisors and the canines and more outward angulation in the lateral incisors.	Good
Iodice et al.; 2013 J Craniofacial Surgery [38]	Prospective study	21 patients; Median age 25.6 ± 6.3 years; TMD > 5 mm	Le Fort I osteotomy + 2-piece osteotomy + Pterygomaxillary disjunction (PD)	Lateral cephalogram before and 6 months after surgery 3 years	skeletal measurements: - SNA (°), - S–N-Sna angle (°), - FH^NA angle (°), - SN^PP angle (°), - FH^PP angle (degrees), - SNB (°), - S–N-Pg angle (°), - SN^GoGn angle (°), - FMA (°), - upper anterior facial height (UAFH)/total anterior facial height (TAFH) (numerical value), - lower anterior facial height (LAFH)/ TAFH (numerical value), - upper posterior facial height (UPFH)/ total posterior facial height (TPFH) (numerical value), - TAFH/TPFH (numerical value), - upper incisor (U1)^NA (°), U1^SN (°), U1^FH (°)	The skeletal vertical and sagittal changes did not reveal any variables with statistically significant results both in the maxilla and in the mandible. The upper incisor changes after the SARPE. showed statistically significant alterations in U1^NA, indicating posterior inclination (*P* = 0.049); the others (U1^SN, U1^FH) confirmed the posterior inclination of the upper incisors but were not statistically significant (*P* = 0.1 and 0.08, respectively).	Fair
Antonios Sygouros et al. 2014 American Journal of Orthodontics and Dentofacial Orthopedics [39]	Retrospective study	20 patients SARME -PD (10 patients; mean age, 19.2 years) SARME 1PD (10 patients; mean age, 18.4 years) TMD	Le Fort I osteotomy + 2-piece osteotomy ± Pterygomaxillary disjunction	CBCT - immediately before surgery - 3 months after surgery - 6 months after completion of the active expansion	Skeletal measurements - Distance between the left and right piriform rims - Distance between jugale points bilaterally - Distance between the lateral pterygoid plates bilaterally - Angles between the left and right lateral pterygoid plates and medial pterygoid plates Dentoalveolar measurements - Distance between the canine furcation points bilaterally - Distance between the first premolar furcation points bilaterally. - Distance between the second premolar furcation points bilaterally. - Distance between the first molar furcation points bilaterally. - Angulation of the alveolar crests. Dental measurements - Intercanine width - Interpremolar width for first premolars - Interpremolar width for second premolars - Intermolar width. - Canine angulation. - First premolar angulation. - Second premolar angulation. - Molar angulation. - Vertical molar movement. Periodontal measurement - Width of the buccal alveolar bone in the canine region. - Width of the buccal alveolar bone in the first premolar region. - Width of the buccal alveolar bone in the second premolar region. - Alveolar bone height in the canine region. - Alveolar bone height in the first premolar region. - Alveolar bone height in the second premolar region. - Alveolar bone height in the first molar region.	Anterior skeletal expansion was evident in both groups with no statistically significant differences between them. At the dentoalveolar level, significant expansion was obtained between contralateral teeth (P0.01), with no significant differences between the 2 groups. More pronounced tipping, without statistical significance, was reported in SARME + PD group. On the dental level, all distances between cusps of the contralateral teeth increased significantly in both groups with no difference between the groups. SARME resulted in buccal tipping of all posterior teethbut not of the canines. On the periodontal level (Table VIII), the width of buccal alveolar bone decreased for all posterior teeth in both groups. The height of the alveolar crest was reduced more in the SARME-PD group in the premolar area, but the difference did not reach statistical significance.	Fair
Habersack et al., 2014 JOMS [40]	Retrospective cohort study	24 patients: 2-piece (*n* = 12) 3-piece (*n* = 12) Mean age: 27 ± 18.5 years TMD > 5 mm	Le Fort I osteotomy + 2-piece or 3-piece osteotomy + Pterygomaxillary disjunction (PD)	Postero-anerior cephalogram - before surgery (T1) - 6 weeks (T2) after surgery, - 6 months (T3) after surgery, - 12 months (T4) after surgery	- Interdental width changes at the intercanine level; - interdental width changes at the level of the second molars; - skeletal expansion between the bilateral maxillary jugulum landmarks.	There was a significant difference in the intercanine distance between the 2-piece and 3-piece SARME groups at T2, T3, and T4 (*P* < .001): 2-piece osteotomy showed higher values at any timings. No significant difference between the 2-piece and 3-piece SARME groups was found in the inter-Second Molar Distancesat (*P* > .05). No significant difference between the 2-piece and 3-piece SARME groups was found in the Skeletal Jugulum Distance (*P* > .05).	Good
Daif 2014 IJOMS [5]	Prospective study	30 patients; Age 20 to 29 years, mean age 24 years; TMD > 5 mm	Le Fort I osteotomy + 2-piece osteotomy - Pterygomaxillary disjunction (PD)	CT - before surgery - 6 months after surgery	Palatine process of the maxilla (C1) Inferior palatine margin of the alveolar process of the maxilla (C2) Greatest convexity of the medial walls of maxillary sinus (A1) greater palatine foramen (A2)	Maxillary expansion was higher at C2 than C1, this indicates tilting of the expanded segment outward at the C2 level Maxillary expansion was higher at A1 than at A2, this indicates tilting of the expanded segment outward at the A1 level No complications;	Good
Adolphs et al.; 2014 Journal of Cranio Maxillo Facial Surgery [41]	Retrospective case series	50 patients Mean age: 24.6 years TMD could not be corrected by orthodontic appliances alone	modified subtotal Le Fort I osteotomy + median maxillary split - pterygomaxillary disjunction	- Photo documentation, - distraction protocols, - rhinomanometry - dental casts - DICOM datasets Follow-up after device removal is more than 6 months	**-**Amount of dental expansion; **-**Complications and relapse; -Pain	Maxillary expansion improved nasal breathing in all patients (28/50) in whom pre- and postoperative rhinomanometry was performed routinely. No palatal relapse consisting in a reduction of the initially achieved widening was observed. In one patient, infraction of the vestibular alveolar process occurred during median splitting of the maxilla.	Low
William Yao et al. 2015 Craniomaxillofacial deformities/cosmetic surgery [42]	Prospective longitudinal study	13 patients MULTIPIECES OSTEOTOMY GROUP (9 patients) SARPE (4 patients) 17 – 23 Years TMD	SARPE GROUP Le Fort I osteotomy + 2-piece osteotomy + Pterygomaxillary disjunction MULTIPIECES OSTEOTOMY GROUP Le Fort I + 2- or 3-piece osteotomy + Pterygomaxillary disjunction	CBCT SARPE GROUP - preoperatively (T0), - after expansion in retention (T1), - at least 6 months after expansion (T2) MULTIPIECES OSTEOTOMY GROUP - preopera-tively (T0), - within 1 month postoperatively (T1), - at least 6 months postoperatively (T2)	**Posterior skeletal width:** measured from the most posterior point of the greater palatine canal in the axial view at the level of the nasal floor, bilaterally **Anterior skeletal width:** measured from the left recess point to the right recess point of the piriform rim **Posterior dentoalveolar width**: from the medial of the left maxillary first molar crown to the medial of the right maxillary first molar crown **Anterior dentoalveolar width:** measured from the medial of the left maxillary canine crown to the medial of the right maxillary canine crown	All patients showed an increase in posterior and anterior skeletal width from T0 to T1. A greater variation in the amount of expansion in the anterior maxilla compared with the posterior maxilla was noted both in the Le Fort group and in the SARPE group. The SARPE group showed a significantly greater amount of dental expansion. A greater increase at the molars respect to the canines was showed in both group from T0 to T1. From T1 to T2, the Le Fort group showed a greater dental relapse at the molars than at the canines.	Low
Seeberger, R. 2010 Journal of Cranio-Maxillo-Facial Surgery [32]	Retrospective clinical mono- centre study	13 patients mean age: 31.23^6.11 TMD ≧ 5 mm	Le Fort I osteotomy + 2-piece osteotomy ± Pterygomaxillary disjunction	Acoustic rhinometry - One month before surgery - 63 months after the operation	Volume of nasal airways divided in the different segments: - Anterior segment of the nasal cavity, - medium segment of the nasal cavity, - posterior segment of the nasal cavity - nasal isthmus	All patients showed a significant enlargement of the nasal volume as result of the palatal transverse distraction. The volumes of the different segments indicate a V-shaped movement of the segments. The gain was greater in the anterior than in the medium and posterior segments. No relapse of the maxillary expansion was observed. Stable orthognathic occlusions were observed 63 months after treatment. All patients reported substantial improvement of nasal respiration.	Good
Dergin et al.; 2015 Oral Surg Oral Med Oral Pathol Oral Radiol [43]	Retrospective clinical study	60 patients; 17–26 years; TMD > 5 mm	Le Fort I osteotomy + 2-piece osteotomy + Pterygomaxillary disjunction (PD)	CT	Assessment of post-operative discomfort and complications after 6 months	Nasal bleeding occurred in 12 of 60 patients (20%); Eleven patients reported paresthesia of the infraorbital nerve and related branches; Seven patients suffered prolonged pain during the distraction procedure. Eight patients suffered from headaches One patient developed a large hematoma in the cheek; One patient complained of tinnitus; Three patients reported excessive lacrimation;	Fair
Zandi et al. 2016 Journal of Cranio-Maxillo-Facial Surgery [44]	Double-blind, historical, controlled, clinical trial	30 patients SARME-PD 15 prospective patients + SARME + PD 15 retrospective patients 15 to 28 years TMD	Le Fort I osteotomy + 2-piece osteotomy ± Pterygomaxillary disjunction	CBCT - before treatment (BT) - immediately after the end of the consolidation period (AT)	Nasal floor width measured at the area of the first premolars, and the first molars (NFW4) Palatal bone width measured at the level of a line connecting the palatal root apex of the first premolars and the first molars (PBW4). The distance between the palatal root apex of the right and left first premolars and first molars (IRD4) The distance between the mesiopalatal cusp tip of the right and left first premolars and of the right and left first molars (ICD4).	No significant difference was observed between the groups. Surgical time from incision to last suture was significantly (*P* < 0.05) shorter in the SARME-PD group. Mean Hyrax opening in the SARME-PD and SARME + PD groups was no statistically significant different. Mild extrusion of an anchor first premolar occurred in a patients in the SARME + PD group. The amount and pattern of maxillary expansion were not significantly different between the SARME-PD and SARME + PD groups. In both the SARME-PD and SARME + PD groups, the greatest expansion occurred at the dental arch, followed by palatal bone and nasal floor level, all the differences were statistically significant (*P* < 0.0001). In both the SARME-PD and SARME + PD groups, the amount of expansion achieved at the first premolar and molar areas was comparable, indicating a parallel widening of maxilla postero-anteriorly.	Fair
Oliveira et al.; 2016 Ijoms [45]	Retrospective study	30 patients: Group 1 (16 patients) Group 2 (14 patients) Mean age: group 1: 30.4 years; group 2: 24.2 years TMD > 5 mm with crossbite	Le Fort I osteotomy + 2-piece osteotomy + Pterygomaxillary disjunction (PD) + Two different maxillary lateral osteotomy: - group 1: lateral osteotomy performed in a horizontal straight fashion; -group 2: lateral osteotomy performed in parallel to the occlusal plane with a step at the zygomatic buttress	CBCT - before surgery (T1), - immediately after expansion (T2), - at 6 months after expansion (T3)	Linear and angular measurements were performed on the coronal slices at the level of the upper first molars and upper pre-molars to determine - nasal floor width, - maxillary width, - distance between the palatal root apices, - distance between the buccal cusps - tooth tipping	No differences were found between the groups in the expansion of dental and skeletal measurements (*P* > 0.05). However, changes over time were significant for all measures (*P* < 0.001). Significant differences between time points T2 and T3 were found for the distance between the palatal root apices in molars, intermolar angle, nasal floor width at the level of premolars, and interpremolar angle. A recurrence of nasal floor width (0.29 mm) was observed between T2 and T3. The distance of the root apices of the first molars increased by 1.28 mm during the retention period (*P* < 0.001). Increases of tooth tipping were observed after expansion of molars and premolars as a result of SARME, with a decrease in these angles was observed between T2 and T3 (*P* < 0.05). The amount of expansion in the cusps of the first molars was similar to that obtained in the cusps of the first premolars (*P* = 0.632), with a parallel expansion occurred when evaluated at the coronal level of the teeth supporting the appliance. Similar results were found for measurements at the level of the palatal root apex (*P* = 0.315). The results revealed greater expansion in the lowest region of the maxilla; these results translate to a tilt of the maxillary segment.	Fair
Jensen et Rodrigo-Domingo.; 2017 Oral Surg Oral Med Oral Pathol Oral Radiol [46]	Retrospective cohort study	20 patients: Releasing of the nasal Septum (*n* = 10), No releasing of the nasal septum (*n* = 10) Releasing of the nasal Septum: mean age 18 years; No releasing of the nasal Septum: mean age 23 years TMD > 5 mm	Le Fort I osteotomy + 2-piece osteotomy + Pterygomaxillary disjunction (PD) ± Release of the nasal septum from the palatal base	CBCT - Immediately postoperatively (T1), - at the end of the distraction phase (T2), - 6 months after SARME (T3)	Using a calliper was measured - the distance (mm) between the contact point of the central incisors, - the distance (mm) between the vestibular cusp tips of the first premolars, - the distance (mm) between the mesiobuccal cusp tips of the first molars. Nasal septum deviation was evaluated by measuring the angle obtained between the traced median reference line and the intersection line between the superior orbital rim.	Moreover, objective evidence of nasal septum deviation was not observed in any of the patients in the two groups. No significant difference in dental expansion was found between the two groups (*P* = 0 .46). No statistically significant differences between groups were found at any time point.	Good
M.Verquin et al. 2017 Clinical Paper Orthognathic Surgery [47]	Retrospective study	55 patients 13 to 47 Years TMD	Le Fort I osteotomy + 2-piece osteotomy + Pterygomaxillary disjunction + Disengagement of the nasal septum	Baseline radiographic and clinical imaging	- Nerve injury - Haemorrhage - Intraoral bleeding - Dental and periodontal problems - Pain - Inadequate/asymmetrical expansion - Other complications	Paresthesia of the infraorbital nerve and related branches was observed in 16 patients. Six patients presented with postoperative bleeding Dental complications were seen in a total of five patients. Four patients suffered from severe postoperative pain at the surgical site. Three cases of asymmetrical expansion were encountered.	Fair
Alves et al.; 2017 IJOMS [48]	Retrospective study	19 patients: group 1 (*n* = 9) group 2 (*n* = 10); Mean age of group 1: 23.1 years (19.5– 29.4 years); mean age group 2: 30.3 years (18.7–39.7 years) TMD > 5 mm;	-Group 1: Le Fort I + alar base cinch -Group 2: subtotal Le Fort I + V-shaped incision at the maxillary midline in the labial frenulum region, without alar base cinch	CBCT - before surgery (T1) - 6 months after expansion (T2)	- superior alar width, - alar base width, - nasal width, - alar angle, - nasal length, - nasal projection, - upper lip length	Mean maxillary expansion had no significant difference between the groups (*P* = 0.64). In both groups, a significant increase in nasal width (superior alar width, alar base width, and nose width) after maxillary expansion was observed (*P* < 0.001). Nasal length and projection had no significant changes Alar angle was significantly different pre- and post-surgery only in group 2 (*P* = 0.013). No statistically significant difference was found between groups, except for the post-operative upper alar width, significantly higher in group 1.	Good
Cakarer et al. 2017 J Stomatol Oral Maxillofac Surg [49]	Retrospective case series	40 patients; 23.67 ± 5.23 bilateral TMD > 5 mm	Le Fort I; PMS was not performed but bilateral nasal osteotomies were created	Clinical examination During the 6 months after surgery	Intra- and postoperative complications	During operation, the osteotome became displaced from the palatal mucosa in three patients. The mucosa was sutured and no postoperative dehiscence was observed. No hemorrhagic problem was noted. One patient experienced epistaxis 5 days after surgery. Hemostasis was attained by applying an anterior nasal pack. One patient developed a maxillary sinus infection 2 weeks after surgery and four complained of numbness of the anterior maxilla. One patient with cleft lip-palate (CLP) syndrome developed a fistula at the site of the prior palatal cleft. One patient exhibited wound dehiscence at the anterior maxilla 1 week after surgery No asymmetric/inadequate expansion, and no dental or periodontal problem was noted in any patient	Fair
Romulo de Medeiros et al.; 2017; International Journal of Oral and Maxillofacial Surgery [50]	**RCT**	25 patients; 17—49 years Skeletal transversal maxillary deficiency > 5 mm, with unilateral or bilateral skeletal crossbite	Le Fort I osteotomy + (with) 2-piece osteotomy + (with) /- (without) Pterygomaxillary disjunction (PD)	CBCT; T1 (preoperatively), T2 (after the activation period post-SARME) T3 (after 6 months of Hyrax screw stabilization)	-Nasal cavity volume (NCV); -Right (RMSV) and left (LMSV) maxillary sinus volume; -Nasopharynx volume (NPV); -Oropharynx volume (OPV); -Oropharynx minimum cross-sectional area (OMCSA)	A statistically significant difference was observed only for NPV (*p* = 0.003), OPV (*p* = 0.007), and OMCSA (*P* = 0.001) in SARME + PD group The sum of the NCV, RMSV, LMSV, NPV, and OPV did not differ significantly between SARME + PD and SARME –PD (*p* = 0.983).	Unclear
Kim et al.; 2018 K Journal of Orthod [51]	Prospective study	61 patients: Control group (*n* = 25) Experimental group (*n* = 36) Mean age: CTR group 22.48 ± 3.81 years; Experimental group 24.50 ± 6.19 Years;	Le Fort I osteotomy (control group) ± segmental osteotomy (experimental group) + Pterygomaxillary disjunction (PD)	CBCT - before (T1) - after surgery (T2) - at the end of treatment (T3)	- Measurement of the skeletal width Measurement of the distance between the right and left greater palatine foramina on a coronal cone-beam computed tomography image - Measurement of the dental width Measurement of the distance between the right and left mesiolingual cusps of the first molars on a coronal cone-beam computed tomography image	The amount of change in the dental width after surgery was small in the control group; a significant expansion was achieved in the experimental group. There was no significant difference in the width at T2 between the two groups; this was maintained up to T3. The amount of change in the skeletal width after surgery was very small in the control group; a significant expansion was achieved in the experimental group. The width at T2 was significantly greater in the experimental group than in the control group. During the postoperative orthodontic treatment period, the experimental group exhibited a significant decrease in the skeletal width (− 0.67 ± 0.72 mm), unlike the control group (0.30 ± 0.87 mm).	Fair
Krzysztof et al.; 2018 [52]	Prospective study	78 patients Mean age: 16.86 ± 2.65; TMD > 4 mm	Le Fort I osteotomy + 2-piece osteotomy + Pterygomaxillary disjunction (PD)	CBCT + Lateral cephalogram - Before - 3 months after surgery	Using gypsum models, measurements in millimetres (mm) were performed: - distance between points on the cusp tips of the canines; - distance between points on the buccal cusp tips of the first maxillary premolars, - distance between points on the cusp tips of the first maxillary molars The following were assessed on CBCT: - distance between points located the most laterally on the internal - bone surface of the nasal cavity at the level of the first maxillary molars - distance between palatal cusps of the first maxillary molars - distance between the skeletal margins from the palatal side of the first maxillary molars - palate height measured at the level of the first maxillary molars	The intercanine dimension, between the cusp tips of the maxillary canines T1-T2 was 7.68 ± 3.78 (*p* < 0.05). The anterior arch width, between the palatal cusp tips of the first maxillary premolars T1-T2 was 8.26 ± 3.08. The posterior arch width, between the palatal cusp tips of the first maxillary premolars T1-T2 was 5.98 ± 2.60. Nose floor did not change significantly after surgery.	Fair
Huizinga et al.; 2018 J Cranio Maxillofacial Surgery [6]	Retrospective case series	20 patients; Mean age: 24.5 years; TMD	Le Fort I osteotomy + 2-piece osteotomy + Pterygomaxillary disjunction (PD)	CBCT and 3D reconstruction of the cone beam computed tomography (CBCT) scans - predistraction model - postdistraction model (5 months after)	Differences in lateral expansion between right and left maxillary segments were calculated in three ways: 1) anteriorly, at the level of the central incisor; 2) posteriorly, at the level of the first molar inferiorly and at the level of the first molar superiorly	Clinical relevant asymmetries (> 3.0 mm) were found in 11 patients (55%). Five patients had an asymmetry of > 5.0 mm in one or two directions. Among asymmetric lateral expansions, most of them occurred in the anterior-inferior component (48.8%). Only 24.1% of asymmetric expansion occurred in the inferior-posterior component. Caudal expansion was larger than cranial one.	Good
Ferraro-Bezerra et al.; 2018; Journal of Oral and Maxillofacial Surgery [53]	**RCT**	24 patients; 17—49 years Skeletal transversal maxillary deficiency > 5 mm, with unilateral or bilateral skeletal crossbite	Le Fort I osteotomy + (with) 2-piece osteotomy + (with) /- (without) Pterygomaxillary disjunction (PD)	CBCT; T0 (preoperatively) T1 (at the end of the expansion) T2 (6 months after the final activation and before the hyrax removal)	- Skeletal and dental expansion at the posterior maxillary region; - Skeletal and dental expansion at the anterior maxillary region; - Molar tipping	In both groups, all of the measurements significantly increased between T0 and T2, except for maxillary width at the molar region. Although without significant difference, between T1 and T2, greater posterior palatine bone expansion was found in SARME + PD group and greater molar dental expansion in SARME -PD group.	Good
Mohlhenrich et al.; 2020 Oral Surg Oral Med Oral Pathol Oral Radiol [54]	Retrospective study	27 patients: SARME + PD (*n* = 15) SARME-PD (*n* = 12) Mean age: SARME + PD (26.3 years); SARME-PD (26.3 years); TMD	Le Fort I osteotomy + 2-piece osteotomy ± Pterygomaxillary disjunction (PD)	Plaster casts before and after surgery Mean follow-up: 24.6 months	The maxillary dental arch width measured as: - distance between the vestibular and palatal cusp tips of the canine and the first and second premolars - distance between the mesiobuccal and mesiopalatal cusp tips of the first and second molars For evaluation of transverse asymmetry, the distance between the right side of the arch width and the raphe palatina line was measured and subtracted from the distance on the left side The palatal gingival depth measured as shortest distance from the midpalate raphe to the connecting line between the gingival crests adjacent to the first molars The palatal vault angle measured a) between the tangential lines to the middle two-thirds of the right and left palatal surfaces b) between the intersecting lines drawn across the mesial buccal and mesial lingual cusp tips of the right and left first molars was measured to determine the axial angulation of the maxillary first and second premolars and the first and second molars	Significant changes in the dental arch width were observed in all pre- and posttreatment transverse measurements. No significant differences between both groups in the mean changes (T1_T0) were found. No significant differences in the expansion pattern were found. An increase in crown height was measured in nearly all teeth in both groups, but this increase was not significant. The increase was less or negative in the group with PMD Statistically significant differences in the pre- and post-treatment conditions were found in the palatal gingival width (C) and midpalate width (D). No significant differences were detected in the treatment difference values (T1 _ T0), although they were descriptively larger in the ( +) PMD group than in the (_) PMD group. Only the difference in the mean overall changes on the oral side of the right alveolar ridge, that is, _1.96 mm (SD 0.72) for (_) PMD and _2.69 mm (SD 1.02) for ( +) PMD, was statistically significant. Almost no changes were measured in the hard palate, about _0.18 mm (SD 0.52) for (_) PMD and _0.07 mm (SD 0.26) for ( +) PMD.	Fair
Keskin et al.; 2020 J Stomat Oral Maxillofac Surg [55]	Prospective study	18 patients; Mean age 15–33 years; TMD > 5 mm	Le Fort I osteotomy + 2-piece osteotomy - Pterygomaxillary disjunction (PD)	CBCT - before surgery - 6 months after surgery	On digital dental records - Arc circumference (AC), - arc length (AL), - arc depth (AD), - intercanine width, - intermolar width On the CBCT - The volume of the area over the ANS-PNS plane located between the lateral nasal wall and the nasal base in the coronal section - The pre-op and post-op minimum transverse diameters of the bony nasolacrimal duct (BNLD-TD)	Lower anterior nasal volume showed statistically significant increase. Significant dental and skeletal changes in CBCT occurred before and after expansion. BNLD-TD showed statistically significant decrease	Fair
Gürsoytrak et al.; 2021 Meandros Med Dental J [56]	Retrospective study	38 patients: SARME + PD (17 patients); SARME-PD (17 patients) Mean age:19.68 TMD > 5 mm	Le Fort I osteotomy + 2-piece osteotomy ± Pterygomaxillary disjunction (PD)	Clinical evaluation	Intraoperative and post-operative 6-month complications were evaluated in the group with and without PMD	Intraoperative hemorrhage: 2 patients in the with PMD group and 1 patient in the without PMD group. In the with PMD group, post-operative hematoma was observed in 2 patients following intraoperative hemorrhage, Post-operative asymmetric expansion in 2 patients of SARME + PD, and post-operative unilateral transient paresthesia in 1 patient In the without PMD group, 1 patient developed epistaxis after intraoperative hemorrhage The average age was found to be higher in the groups with complications	Good
Da Costa et al. 2021 JOMS [57]	Retrospective study	61 patients: 3-piece (*n* = 32) 2-piece (*n* = 29); mean age 24.5 y; 2-piece: moderate TMD; 3-piece: moderate to large TMD with unilateral or bilateral cross bite	Le Fort I osteotomy + 2-piece or 3-piece osteotomy + Pterygomaxillary disjunction (PD)	CBCT - 1 week postoperatively, - 6 months postoperatively, - 1 year postoperatively	- Skeletal expansion (distance between the right and left maxillary points); - Dental expansion (width between the most buccal points of the maxillary molars); - Pain; - Postoperative complications; - Relapse;	No statistical differences in skeletal maxillary expansion (*P* = .775) between the 2 treatment groups; Significantly more dental expansion following SARPE (*P* = .009); Complications: 3 patients (9.4%) showed bone resorption following 3-piece osteotomy; tooth discoloration and gingival recession was present in respectively 2 (4.8%) and 1 (2.4%) patient of the 2-stage treatment; severe apical resorption was reported in 53.1% of the 2-piece and 66.7% of the 3-piece. This difference was not significant (*P* = 0 .311). Asymmetrical expansion was noticed in 2 patients (4.8%) after the SARPE procedure. Three patients (9.4%) reported mucosal or maxillary sinus infection after 3-piece and 4 patients (12.5%) underwent removal of osteosynthesis hardware due to infection Pain was significantly more reported in the 2-piece group (*P* = .038) Relapse: unilateral posterior crossbite in 2 patients (6.2%) in the 3-piece and 2 patients (4.8%) in the 2-piece at 1 year postoperatively	Fair
Prado et al.; 2021; Journal of the American Society of Plastic Surgeons [58]	**RCT**	32 patients; 16—50 years Transversal maxillary deficiency greater than or equal to 5 mm	Le Fort I osteotomy + (with) 2-piece or 3-piece osteotomy + (with) Pterygomaxillary disjunction	TC and Facial Scanning T0: before SARME T1: end of maxillary expansion T2: removal of expanding device T3: 6 months after T2 T4: 10 months after T1	-Asymmetry of maxillary expansion; -Stability of the area and volume of the palate after expansion; -Changes in nose width and paranasal region	Two-piece osteotomy produced a larger mean increase in nose width (2.73 mm); however, 3-piece technique produced a larger displacement of the paranasal areas (*p* = 0.014) The symmetry of maxillary expansion did not differ between the 2-piece or 3-piece osteotomies. Relapse of palatal area and volume was similar in the two study groups	Fair
Pereira et al. 2022 Oral Surgery, Oral Medicine, Oral Pathology and Oral Radiology [59]	Pilot prospective study	19 patients: 10 2-S osteotomy 9 3-S osteotomy Mean age: 25.7 years (18–33 years) TMD ≧ 6.0 mm	Le Fort I osteotomy + 2-piece or 3-piece osteotomy + Pterygomaxillary disjunction	CBCT - before surgery - after expansion completion, - 2 months after surgery	Palatal expansion: - distance (mm) between the bone segments bordering the osteotomy gap in 2-piece and the sum of the 2 distances between the bone segments bordering the 2 osteotomy gaps in 3-piece; - distance (mm) between the mesio-palatal cusp tips of the maxillary first molars; - the angle formed between a line connecting the apex of the maxillary first molar’s palatine root with the mesio-palatal cusp, and a line perpendicular to the horizontal plane The interradicular space: - distance between the central incisors in 2-piece and between the lateral incisors and canines in group 3-piece Periodontal probing: performed immediately before surgery and 2 months after maxillary expansions on buccal, palatal, mesial, and distal surfaces of the teeth adjacent to the osteotomy Cosmetic perception: using a colored visual analog scale (VAS), in which 0 was the worst aesthetic result and 10 was the best	The 3-piece osteotomy had statistically significant (*P* = .016) values of increased bone expansion The mean increase in the distance between the tips of the mesiopalatal cusps of the maxillary first molars was greater in the 3-piece, with no significant differences between them The first molar had greater bucco-palatine angulation values in the 2-piece, with significance only on the right side (*P* = 0.028) In the 2-piece test, patients were clearly dissatisfied compared with the 3-piece test, the mean score was 7.68 ± 1.77 (*P* = 0.000) The periodontal survey revealed that 100% of postoperative measurements remained compatible with periodontal health	Good
Orion et al. 2022 J Cranio Maxillo-Fac Surg [60]	Prospective study	11 patients Mean age of 38.89 years TMD	MISMARPE: Le Fort I osteotomy + 2-piece osteotomy - Pterygomaxillary disjunction (PD)	CBCT - before expansion (T0) - at the end of the activation period (T1)	(D1): Posterior maxilla distance; (D2): Posterior midpalatal suture distance; (D3): Anterior maxilla distance; (D4): Anterior midpalatal suture distance; (D5): Posterior alveolar process distance; (D6): Anterior alveolar process distance; (D7): Posterior dental crown distance; (D8): Posterior dental root distance; (A1): Angle UR6; (A2): Angle UL6	Greater expansion was observed in the anterior region than in the posterior region In the posterior region, a greater expansion was seen in the maxilla than in the alveolar process	Fair
Felipe et al.; 2022 Research, Society and Development [61]	Retrospective and observational study	19 patients; SARME + PD (6 patients); SARME – PD (13 patients) Mean age: 28.94 ± 8.38 years TMD	Le Fort I osteotomy + 2-piece osteotomy ± Pterygomaxillary disjunction	CBCT - one month before surgery (T0) - six to eight months after surgery (T1)	On a plaster model - the distance from the palatal cusp of the right first molar to the palatal cusp of the left first molar On the CBCT: - the thickness of buccal alveolar bone and the height of buccal alveolar bone were measured in the upper canines, maxillary first and second premolar, and maxillary first molar - the angle between the alveolus and the long axis of the tooth	In each tooth and on both sides of the maxilla, there were no significant differences in the variables between groups SARME + PD and SARME-PD On the left side, there were differences between the group with PMD and the group without PMD, where the second premolar and first molar showed increased angulation of the alveolus The greater the amount of expansion, the greater the bone level and the thickness loss	Fair
Carvalho et al. 2023 Oral and Maxillofacial Surgery [62]	Retrospective observational study	24 patients: SARME + PD (*n* = 13); SARME-PD (*n* = 11) mean age 27.2 ± 1.6 NA	Le Fort I osteotomy + 2-piece osteotomy ± Pterygomaxillary disjunction (PD)	CBCT - Before surgery(T1) - Postsurgery (T2), after the activation period)- Postsurgery (T3), 6 months after Hyrax screw stabilization	Sagittal and vertical maxilla-mandibular measurements after SARME using Linear and angular cephalometric parameters	Significant maxillary displacements in the right-left component in both groups Regarding the anteroposterior orientation, a significant reduction was observed in the PD group at T3 (*p* = 0.022) Craniocaudal N-Pg (*p* = 0.018) and N-Pg (3D) (*p* = 0.016) varied significantly at T2 and returned to baseline at T3 in the PD group	Good

*CBCT* Cone Beam Computed Tomography, *SARME* Surgically Assisted Rapid Maxillary Expansion, *CT* computed tomography

### Study characteristics

Twelve studies were conducted in Brazil, nine studies in Germany, six in Turkey, three in Belgium, two in Italy, and the remaining eight studies were conducted differently in USA, Iran, Denmark, The Netherdlands, Egypt, Spain, Poland, and Korea [5, 6, 26, 27, 29–37, 39–48, 50, 51, 54–59, 61–68]. All the studies were published between 2001 and 2023 [5, 6, 26, 27, 29–37, 39–48, 50, 51, 54–59, 61–68].

A total of 1513 adult patients with transverse maxillary deficiency greater than or equal to 5 mm, with a mono- or bilateral crossbite, were treated under general anesthesia [5, 6, 26, 27, 29–37, 39–48, 50, 51, 54–59, 61–68]. In relation to the segmental surgical procedure, Le Fort I osteotomy was performed in association with two different osteotomies: separation of midpalatal suture (2S; 1196 patients), or bilateral vertical osteotomy running between the lateral incisor and the canine from the piriform aperture to the alveolar crest (3S; 146 patients) [5, 6, 26–28, 30, 32, 33, 36–41, 43–48, 50, 51, 54–59, 61–63, 65, 67, 68]. According to posterior osteotomy, Le Fort I was performed with or without the disjunction of pterygomaxillary suture. PD was performed in 1128 patients, while 558 patients did not record this osteotomy [5, 6, 26–28, 31, 33, 34, 37–41, 43–48, 50, 51, 54–59, 61–63, 65–68]. Four studies (121 patients) failed to report detailed information concerning the number of patients who received the different osteotomies [31, 32, 42]. Eleven studies directly compared SARME + PD and SARME-PD [31, 32, 36, 39, 44, 50, 54, 56, 61–63]. Eight studies specifically examined the comparison between 2-piece and 3-piece osteotomy [29, 31, 36, 37, 40, 57–59].

Orthodontic treatment was performed with tooth-borne distractors (TB) in 1006 patients and with a bone-borne distractors (BB) in 149 patients [5, 6, 26–28, 30, 32–34, 38–40, 42–45, 47, 48, 50, 54–59, 61–63, 65–68]. Five studies not described how many patients received TB or bone-borne distractors (BB), Kim et al. conducted a study analyzing palatal expansion following segmental Le Fort I osteotomy fixed using osteosynthesis plates and screws, so any additional orthodontic force was not applied [29, 31, 36, 37, 46, 51]. The TB device was located by the treating orthodontist preoperatively. BB distractors were applicated intraoperatively at the second premolar level.

A Computed Tomography (CBCT) was performed preoperatively and postoperatively for most of all subjects [5, 6, 28, 29, 31, 36, 37, 39, 42–46, 48, 50, 51, 55, 57–59, 61, 63, 65, 66, 68]. The other patients underwent to baseline radiograph and/or cephalogram, plaster cast, acoustic rhinometry measurement, and clinical examination [26, 27, 30, 33, 34, 38, 40, 47, 54, 56, 67].

Quantitative analysis of the included studies could not be performed, considering the large difference in the outcomes’ assessment.

### Assessment of quality

**Fig. 2:**
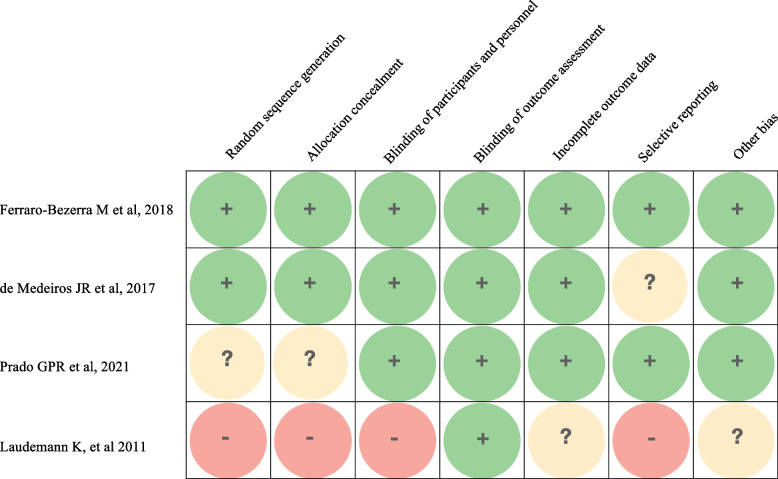
Risk of bias assessment for the included RCTs

About the RCTs, the analysis of the methodological quality showed a low risk of bias in two articles [58, 63]. One study reported a high risk of bias [36]. An unclear risk of bias was recorded for one RCT [50]. The most critical items of the Cochrane Collaboration Tool were in the selection bias and reporting bias (Fig. 2). Among the non-RCTs, 15 studies showed a good level of quality, while 21 exhibited a fair quality score (Table 2) [5, 6, 26, 27, 29–35, 37, 39–48, 51, 54–57, 59, 61–68].

**Table 2:** Newcastle–Ottawa quality assessment

Author, year	Newcastle–Ottawa scale outcome (range 0–9)	Quality assessment
1. Matteini, 2001 [26]	5	Fair
2. Babacan, 2006 [27]	6	Good
3. Goldenberg, 2007 [28]	5	Fair
4. Landes, 2009 [29]	6	Good
5. Marchetti, 2009 [30]	6	Good
6. Laudemann, 2009 [31]	6	Good
7. Seeberger, 2010 [32]	6	Good
8. Alfaro, 2010 [33]	3	Fair
9. De Assis, 2010 [34]	6	Good
10. Landim, 2011 [35]	5	Fair
11. Landes, 2012 [37]	6	Good
12. Iodice, 2013 [38]	5	Fair
13. Sygouros, 2014 [39]	4	Fair
14. Habersack, 2014 [40]	7	Good
15. Daif, 2014 [5]	5	Fair
16. Adolph,2012 [41]	3	Fair
17. Yao, 2015 [42]	4	Fair
18. Seeberger, 2015 [66]	6	Good
19. Dergin, 2015 [43]	3	Fair
20. Zandi, 2016 [44]	5	Fair
21. Oliveira, 2016 [45]	5	Fair
22. Jensen, 2017 [46]	6	Good
23. Verquin, 2017 [47]	5	Fair
24. Alves, 2017 [48]	6	Good
25. Cakarer, 2017 [67]	4	Fair
26. Kim, 2018 [51]	5	Fair
27. Krzysztof, 2018	5	Fair
28. Huizinga, 2018 [6]	6	Good
29. Möhlhenrich, 2020 [54]	5	Fair
30. Keskin-Yalcin, 2020 [55]	4	Fair
31. Gursoytrak, 2021 [56]	6	Good
32. Da Costa Senior, 2021 [57]	5	Fair
33. Pereira, 2022 [59]	6	Good
34. Orion, 2022 [60]	4	Fair
35. Felipe, 2022 [61]	5	Fair
36. Carvalho, 2023 [62]	6	Good

### Study outcomes

#### Skeletal outcomes

##### Pterygomaxillary disjunction

All studies reported a significant transversal increase in the maxilla [5, 6, 31, 36, 38, 45, 54, 58, 61–63, 65]. According to pterygomaxillary disjunction, a more homogeneous maxillary expansion is achieved in SARME + PD [5, 36, 58, 61, 63]. The anterior maxillary segment recorded a higher expansion in SARME -PD, confirming the V-shaped sutures opening, showing a posterior expansion lesser than 0.5 mm compared to SARME + PD [36, 54, 58, 63]. SARME-PD showed a differential expansion in the anterior and posterior part of the maxilla up to 3 mm [5]. Greater expansion was observed at the anterior region than the posterior region [65]. However, Lauderman and colleagues stated that PD seemed to have an effective role in patients older than 20 years, while adjunct benefits of this osteotomy were not analyzed in younger subjects [36]. Craniocaudal analysis showed a greater expansion in the alveolar bone than in the palatine level, with a difference up to 2 mm [5, 6, 45, 54, 65]. Immediately after the activation period, the SARME + PD group showed a significant increase in the height of the middle and lower third of the face; however, these changes cancelled out during the stabilization phase, leading to a return to the initial condition [62]. Concerning the direction of the bone movement, anteriorly to posteriorly there was an outward segmental inclination for SARME + PD and an inward one for SARME -PD [31, 36]. Asymmetric expansion could be achieved with a clinically relevant difference between right and left side (> 3 mm), more the anterior-inferior component than in the inferior-posterior component [6]. In terms of transverse widening, bone-borne appliances showed a lower opening than tooth-borne devices but with a lower buccal tipping of the molars and an increased buccal bone plate at premolars, after both SARME + PD and –PD [36].

##### Segmental Le Fort I osteotomy

According to segmental technique, eight studies described the skeletal outcomes between two-segments and three-segments osteotomies [36, 58]. No statistically significant difference between 2 and 3S was found in the asymmetry of the maxillary expansion [36, 40, 57, 58]. However, 3SO showed 1–2 mm of more transverse increase compared to 2SO and expanded more symmetrically [37, 51, 59]. Also, Lauderman et al. reported a significative transverse widening higher than 2 mm in SARME + PD and segmental 3-piece osteotomy [36]. During the postoperative orthodontic treatment period, the 2S group exhibited a significant decrease in the skeletal width [29, 51]. The greatest loss in vestibular bone substance was detected in the bipartite [29]. Concerning the direction of the bone movement, a lateral bending was analyzed in SARME with 2S, while a medial bending was more frequent in 3-piece osteotomy [36].

#### Dentoalveolar outcomes

##### Pterygomaxillary disjunction

Dental transformations were observed in CBCT images comparing prior to and following expansion, with an average expansion ranging from 6 to 9 mm [27, 33, 54, 55, 68]. According to pterygomaxillary disjunction, dental tipping occurred preeminently in SARME -PD group. SARME + PD group showed a lower tendency of buccal molar tipping (≅ − 2 degrees) either after appliance removal or after 6 months follow-up [63]. The major molar tipping in SARME -PD determined a molar expansion greater than 1 mm compared to SARME + PD, especially with a tooth-borne device. As reported by Laudemann and colleagues, dental tipping showed an outward tipping in TB devices and an inward tipping in BB devices, according to the different level of distraction at which the two devices act (BB at bone level; TB at dental level) [36]. Most teeth in both groups ( SARME + PD and SARME –PD) exhibited a rise in crown height, though with not statistical significance [54]. In SARME –PD group the dental expansion was higher than SARME + PD group, with a decreased degree of canine inclination when PD was not performed. However, when PD was performed, dental expansion was more in the anterior palatal region than in the posterior arch width [68]. The SARME + PD group showed an increased but nonsignificant vestibular bone resorption in the second molar region and an increased palatal bone plate thickness on the first premolar. In contrast, at the premolar level a higher bone resorption was found in SARME-PD and a greater increase in vestibular bone plate thickness was decorded in the SARME + PD group when bone-borne devices were used [31]. In terms of stability, Ferraro-Bezerra M. et al. assessed an increased distance between the buccal and palatal alveolar processes during the retention period [63].

##### Segmental Le Fort I osteotomy

According to segmental technique, six studies reported the dentoalveolar outcomes between two-segments and three-segments osteotomies. Significantly more dental expansion was noted following 2-piece than 3-piece (*P* = 0.009) [40, 51, 57]. Although the difference in the increase in the distance between the mesiopalatal cusps of the maxillary first molars was not significant between two groups (2S and 3S), a greater variation in the angulation of the first molar was found in the 2-piece group [59]. Intermolar distance was found to increase by 2.5–7.5 mm when bipartite Le Fort was performed, while at the intercanine level this increase was less [30]. An augmented dental tipping was found in the 3-piece osteotomy, with a greater inward angulation for the central incisors and canines, while the lateral incisors went to a greater outward angulation [37]. The different position of the post-operative diastema (between the central incisors in 2S; between the lateral incisors and the canines in 3S) showed a better psychological impact for patients undergoing to SARME with segmental 3-piece Le Fort I [58]. The patients’ perceptions of smile aesthetics after expansion were significantly different: the 2S group were clearly dissatisfied compared with 3S group [59]. In assessing periodontal status, periodontal probing revealed that 100% of the postoperative measurements remained between 1 and 2 mm for the whole sample. Because this interval is totally compatible with periodontal health, no statistical test was deemed necessary to evaluate differences between both groups [59].

#### Upper airway outcomes

##### Pterygomaxillary disjunction

According to PD, maxillary expansion improved nasal width and breathing [27, 34, 41, 48, 55]. In their study, Romulo et al. performed several measurements (minimum cross-sectional area of the oropharynx (OMCSA), nasal cavity volume (NCV), sinus volume (MSV), nasopharynx volume (NPV), oropharynx volume (OPV), and total volume) before and after surgery in order to assess changes in upper airway volume. No statistically significant differences were recorded in the accumulated volume of the upper airways, although + PD group always showed higher values than -PD group at each follow-up [50]. The analysis of the specific items showed that NPV and OMCSA were significantly higher in + PD group both after active expansion and after 6 months follow-up compared to the initial condition (*p* < 0.05). The OPV increased significantly in the + PD group compared to controls, but only after the active expansion follow-up (*p* = 0.007). Osteotomy with PD showed a significant increase in the post-operative alar base, maintained until 36 months after surgery [34, 48]. In patients treated with PD, no statistically differences were detected in the distances from the base of the pyriform aperture to the nasal septum between sides and in the position of the nasal septum in relation to the left and right lateral walls of the pyriform aperture [46].

##### Segmental Le Fort I osteotomy

According to segmental osteotomies, Prado and colleagues showed an increased width of the nose in two- segments than three-segments technique, with a significant difference (*p* = 0.016). Furthermore, the superimposition of the facial scans showed a significantly larger transversal displacement of the paranasal area when horizontal osteotomy with the three-segment technique was performed (*p* = 0.014). No differences on vertical or anteroposterior axes were analyzed.

### Complications and relapse

Only five studies reported data on post-operative relapse [30, 37, 57, 58, 63]. According to PD, Ferraro-Bezerra et al. reported both dental and bone relapse in the anterior region during the retention period, either in SARME + PD or in SARME-PD [63]. Comparing two- and three-segments osteotomies, Prado et al. performed a reintervention in two patients, one for each group, because of an unresolved asymmetrical expansion, however in their study in relation to skeletal stability, no relapse was assessed comparing area and volume of the palate vault both after removal of the expander and after follow-up 6 months; Da Costa reported relapse included unilateral posterior crossbite in 2 patients (6.2%) in the 3-piece and 2 patients (4.8%) in the 2-piece at 1 year postoperatively [57, 58]. Marchetti et al. found minimal recurrence in the intermolar distance when the bipartite Le Fort was performed, while at the intercanine level this increase was found to be smaller and with a higher recurrence rate [30]. Transverse widening was associated with greater decline when 3-piece with pterygoid osteotomy was performed, and a bone-borne device was used [36, 37]. Because of infection, 9,4% of patients treated by Da Costa underwent a reintervention to remove osteosynthesis device. An asymmetric expansion occurred in three cases who required re-intervention by Alfaro et al., 2 patients of SARME + PD treated by Gursoytrak and 2 patients treated by Da Costa, 2 2SO cases by Landes [37, 56, 57].

Reported intraoperative complications included an osteotome-induced palatal mucosal laceration, which was sutured without postoperative dehiscence; two patients developed intraoperative hemorrhage with subsequent hematoma formation in the SARME + PD group; in the group without pterygoid osteotomy, only one patient had hemorrhage [56, 67]. Post-surgical complications were reported as hemorrhage and hematoma in two patients and infraorbital nerve paresthesia in four patients after SARME-PD, one patient and 12 patients, respectively, in SARME + PD group [43, 56, 67].

Sinus infections were reported by Cakarer when pterygomaxillary disjunction was not performed and by Da Costa in SARME + PD cases [57, 67]. Regarding bi- and tripartite osteotomies Landes and Da Costa documented cases of dental damage as discoloration [29, 37, 57]. Pain was more prevalent in the 2-piece group, while complications such as bone resorption and gingival recessions were found in the 3-piece osteotomies [57]. Complications often associated with smoking or noncompliance were noted, such as dehiscence, fistula, pain, headache, and distractor loosening.

## Discussion

The purpose of this study was to systematically review the available randomized clinical trials concerning the different osteotomies that can be performed in surgically assisted rapid maxillary expansion. The analysis focused on skeletal changes, dentoalveolar modifications, upper airway variations, relapse, and complications to evaluate the best outcomes of this surgical procedure.

An ideal therapeutic approach for SARME should achieve the advantages of an efficient maxillary expansion, limiting the intraoperative and post-operative complications [69]. An adequate balance between surgical goals and orthodontic aims is needed. Surgically, SARME should be performed with a simple technique, avoiding successive complications. From an orthodontic viewpoint, SARME should be effective in the transversal orthopedic correction, with a symmetric result, avoiding a post-therapeutic relapse or a periodontal damage of the upper teeth [64, 70]. So, the planned osteotomies should obtain the best result, minimizing the risks. The anatomical regions of major resistance for maxillary expansion are well known, including the posterior processes of the maxillary bone that articulate with the pterygoid processes of the sphenoid bone in a fixed joint, and the median suture of the palate that is a fixed linker between the right and left maxillary bones [71]. On these perspectives, most authors concentrated their attention on the role of pterygomaxillary disjunction and median palatal osteotomy during SARME [44, 59, 71–73]. To summarize the evidence-based results, this review 1) analyzed the outcomes of SARME with or without PD, and 2) compared the clinical findings performing a segmental Le Fort I in 2-piece (2S) or 3-piece (3S) osteotomy.

Despite the different approach, all osteotomies allowed to achieve a correction of the transversal maxillary deficiency both in the short- and long- term follow-up [5, 6, 26, 27, 29–37, 39–48, 50, 51, 54–59, 61–68]. With reasonable evidence, SARME + PD showed a more homogeneous skeletal expansion and, although without a significant difference, the anterior segments of the maxillary bone showed a greater transversal movement [58, 63]. Pterygomaxillary disjunction determined a greater posterior skeletal expansion compared to SARME -PD in which a V-shaped suture opening occurred with higher expansion in the anterior maxillary segment [58, 63]. As reported by Ferraro-Bezerra, at the long-term follow-up, palatal expansion at the molar region exceeded of 0.5 mm in SARME + PD compared to SARME -PD, despite the hyrax opening was lower than 0.4 mm in SARME + PD group [63]. According to the biomechanical statement, the center of resistance located at the posterior part of the median palatal suture allowed a more parallel expansion when PD occurred because the increased expansion at the molar region was mainly related to the lateral rotation of the maxilla associated to the transverse movement [71]. Alternatively, without pterygomaxillary separation, an inhomogeneous bone resistance existed and the V-shape opening pattern was induced by a less impediment at the anterior segments [74]. A recent final element analysis stated that SARME + PD achieved a stable expansion, allowing a uniform dissipation of the distraction stresses in the craniofacial skeleton [6, 7, 47, 62, 75, 76]. As reported by several authors, pterygomaxillary disjunction probably does not interfere with the maxillary expansion, but it can influence the opening pattern [64, 69, 75]. Even in the surgical approach of minimally invasive SARME, Perepérez et al. concluded that the transverse improvement at the pterygoid plates is directly related to an adequate PD [77]. In terms of dentoalveolar changes, there was high evidence that a molar expansion greater than 1 mm occurred in SARME -PD compared to SARME + PD [63]. This clinical finding should be interpretated considering the lower tendency of buccal molar tipping (≅ − 2 degrees) in SARME + PD, either after appliance removal or after 6 months follow-up [63]. These outcomes are in accordance with several authors who described a greater expansion amount in SARME -PD, also recording a greater tendency to dental movement with the negative consequences of instability and periodontal damage [14, 74, 78, 79]. In a finite element study, De Assis et al. were able to show that the bone tension on the molars was greater when PD was not performed, and it could affect the buccal plate resorption [80]. Although with high evidence, an increased buccal bone plate at premolars was found using bone-borne appliances than tooth-borne devices, after both SARME + PD and –PD [36]. Bone-borne devices slightly decrease the tension at the molars, can limit the dental inclination, and can avoid the negative sequelae on the periodontium [74]. However, the recorded buccal tipping could be the result of skeletal and dental effects [77, 81]. Not only the pure dental movement, but also the alveolar bone inclination due to the rotation of the maxillary segments affect the final inclination of the molars and its consequences on the adjacent tissues [77]. Concerning dentoalveolar changes of the anterior teeth, high evidence showed that the amount of the anterior expansion was higher in SARME –PD than SARME + PD, according to skeletal outcomes of a V-shaped opening pattern [63]. However, the intercanine transversal widening decreased between the short-and long-term follow-up, both for SARME + PD and –PD [63]. In this review, the amount of relapse was around 1.5 mm, according to recent systematic review by Gogna and colleagues investigated the stability of SARME correction, concluding that intercanine and intermolar relapse ranged between 0.1–2.3 mm and 0.23–3 mm, respectively [72]. Romano et al. measured 0.5 mm of relapse comparing CBCT before surgery, immediately after expansion, and after 6 months of follow-up, but a lack of long-term follow-up could influence their results [74]. As reported in literature, after SARME it is difficult to determine whether the increase in transversal dimension is effectively due to bone widening rather than to dental movements, although a clinical improvement of breathing could be linked to skeletal effects [77, 81]. According to other authors, in this review no significant difference was recorded comparing nasal volume with or without PD. It’s also common to detect a lower volume at the short-term follow-up compared to initial timing, considering the hemosinus induced by the surgical treatment and the expansion protocol [74, 82]. However, with moderate evidence, a higher NPV and OMCSA were found when PD was performed, both at short- and long-term follow-up, while the OPV increased significantly only after the active expansion [58, 63]. As reported by Nada et al., NPV benefits of a posterior volumetric increase at the horizontal plates of the palatine bones, occurring mainly when SARME + PD was performed [83]. After removing the appliance, the decrease of OPV could be related to the tension of soft tissues, the repositioning of the tongue, and the lower reposition of the palatine plane that allows a consequent improvement of NPV. Despite the upper airway changes, to date no authors recorded a correlation between the different osteotomies of SARME and the increased airflow during breathing, considering this procedure more for orthognathic aims rather than for respiratory purpose [74, 77, 84].

The investigation of the best osteotomy of SARME induced also to summarize data comparing Le Fort I osteotomy with median palatal osteotomy (2S) or with two paramedian palatal osteotomies (3S). According to the segmental approach, maxillary expansion showed limited differences between 2 and 3S in terms of symmetrical pattern of opening. In contrast to these results, some studies recorded better outcomes for three-segment technique [40, 84–86]. Landes and collegues aimed to analyze pre- and post-operative CT of 50 patients, comparing the expansion after SARME with 2S and 3S [37]. No parallel expansion pattern was achieved, but 3-piece osteotomy showed more symmetric opening than bipartite technique. However, no authors reported details concerning the cut-off value beyond which an expansion should be considered unbalanced, and, like the concept of asymmetry, it should be recognized when exceeding the clinical perception [58]. Despite a longer surgical treatment and a higher learning curve, Pereira et al. listed significative advantages for SARME with 3S, including greater transverse bone expansion, lower molar tipping, and lower aesthetic involvement [84]. With low evidence quality, this review agreed with these results, describing a difference in terms of amount of expansion (≅ 3,5 mm TB; ≅ 0,3 mm BB) with an increased transversal width in SARME + PD and segmental 3-piece osteotomy [36]. Concerning the direction of the bone movement, a lateral bending was analyzed in SARME with 2S, while a medial bending was observed in 3-piece osteotomy [36]. The medial bending of the 3-piece osteotomy could limit molar inclination and periodontal complication, especially when an accurate pre-operative evaluation of the bone amount for osteotomy has been done [84, 87, 88]. Undoubtedly, the most evident advantages of SARME with 3S were aesthetic findings, especially when incisal crowding and periodontal damage occurred. Three-segment approach avoids the increase of the nose width and the interincisal diastema that could interfere with psychological well-being and social relationships [37, 40, 84–86]. Despite the immediate benefits of SARME with 3S, it’s important to highlight that this review did not record any differences in relation to skeletal stability at a long-term follow-up. With high evidence, no relapse was assessed comparing area and volume of the palate vault both in 2S and 3S surgery [58]. According to these results, Senior and colleagues didn’t find any difference in terms of transversal stability and complications rate between the two surgical techniques at least one-year follow-up [57].

The assessment of the best osteotomy for SARME should consider details about relapse and complications in order to define the best amount of expansion with limited side effects [58, 63]. According to some studies, meticulous release of all areas of increased resistance during SARME might be associated with increased postoperative discomfort [89, 90]. This surgical approach probably reduces the rate of asymmetric and inadequate expansion [20, 43, 47]. In a study with 10 months follow-up, skeletal stability after maxillary expansion was ensured when the pterygomaxillary disjunction was performed [91]. However, it’s mandatory to highlight that this approach is more invasive and it can be associated with post-operative consequences. The studies included in this systematic review reported few data on transversal relapse according to the different surgical technique. Although some clinical studies did not record any significant differences according to specific techniques, low evidence of results doesn’t allow to achieve adequate conclusions. It’s advisable that future RCTs concentrate their attention on this aspect that can influence the surgical choice.

The findings of this systematic review underscore the notable stability achieved in maxillary expansion through pterygomaxillary disjunction, highlighting its central role in the clinical practice of SARME. Furthermore, the 3-piece osteotomy could be considered as crucial factor in achieving substantial aesthetic enhancements in SARME procedures.

In conclusion, the results of this systematic review should be critically analyzed. The existing moderate evidence was based on a limited number of randomized controlled trials (RCTs) within the included studies of this review. Moreover, the heterogeneity among the incorporated trials precluded a quantitative evaluation with a meta-analysis, due to the variations in result assessment. Clinical relevance was highlighted in performing pterygomaxillary disjunction to achieve more stable and homogeneous maxillary expansion with fewer side effects. In terms of segmental osteotomy, 3-piece Le Fort I might be preferred for greater expansion, limiting lateral bending of bone segments and achieving a better esthetic result. More studies are needed to confirm these results and additional research should involve randomized clinical trials to enhance methodological designs, meticulous randomization protocols, and the generation of more objective outcomes.

### Supplementary Information

**Additional file 1: Supplementary Table 1.** List of the excluded studies.

## Data Availability

No datasets were generated or analysed during the current study.
