# Supplementary material for: Can different osteotomies have an influence on surgically assisted rapid maxillary expansion? A systematic review
Source: Head Face Med. 2024 Mar 8;20:16. doi: 10.1186/s13005-024-00415-3 (PMC10921779; doi:10.1186/s13005-024-00415-3)
Supplement: Supplementary file 1 — Additional file 1: Supplementary Table 1. List of the excluded studies. [file 13005_2024_415_MOESM1_ESM.docx]

**Supplementary Table 1 -** List of the excluded studies

| **Title** | **Author** | **Year** | **Reason of exclusion** |
| --- | --- | --- | --- |
| Le-Fort-I advancement with segmental palatal osteotomies in patients with cleft palates | TIDEMAN, H and STOELINGA, P and GALLIA, L | 1980 | Other topic |
| Long-term stability after inferior maxillary repositioning by miniplate fixation | BAKER, DL and STOELINGA, PJW and BLIJDORP, PA and BROUNS, JJA | 1992 | Other topic |
| The use of position screws as an alternative to plating in segmental Le-Fort-I osteotomies | ROCHE, YA and SCHWARTZ, HC | 1994 | Other topic |
| Cleft-orthognathic surgery - complications and long-term results | POSNICK, JC and TOMPSON, B | 1995 | Other topic |
| Sensory nerve disturbance following Le Fort I osteotomy | Aldin, OFS and Coghlan, KM and Magennis, P | 1996 | Other topic |
| Long-term results of segmental repositioning of the maxilla in cleft palate patients without previously grafted alveolo-palatal clefts | Erbe, M and Stoelinga, PJW and Leenen, RJ | 1996 | Other topic |
| Multiple segment osteotomy (MSO) in maxillofacial surgery | Chen, YR and Yeow, V | 1997 | Other topic |
| Multiple-segment osteotomy in maxillofacial surgery | Muchen, YR and Yeow, VKL | 1999 | Other topic |
| Combining single- and double-tooth osteotomies with traditional  Orthognathic surgery | Yeow, VKL and Chen, YR and Su, CP | 1999 | Other topic |
| Effect of segmental Le Fort I osteotomy on maxillary tooth type-related pulpal blood-flow characteristics | Emshoff, R and Kranewitter, R and Gerhard, S and Norer, B and Hell, B | 2000 | Other topic |
| Open bite: stability after bimaxillary surgery - 2-year treatment Outcomes in 58 patients | Fischer, K and von Konow, L and Brattstrom, V | 2000 | Other topic |
| [Treatment of a Class III anterior open bite malocclusion: a combined orthodontic and orthognathic surgical approach.](https://www.google.com/url?q=https://www.google.com/url?q%3Dhttps://pubmed.ncbi.nlm.nih.gov/11699351/%26source%3Dgmail-imap%26ust%3D1685480936000000%26usg%3DAOvVaw3Ze5NtYSRDE3COC3pnZcIX&source=gmail-imap&ust=1694334883000000&usg=AOvVaw0qhEVhult84r4DNlwVRdhv) | Ong HB. | 2001 | Other topic |
| Predicting upper lip response to 4-piece maxillary lefort I osteotomy | Brooks, BW and Buschang, PH and Bates, JD and Adams, TB and English, JD | 2001 | Other topic |
| Treatment of severe mandibular prognathism in combination with maxillary Hypoplasia: case report | Ogasawara, T and Kitagawa, Y and Ogawa, T and Yamada, T and Nakamura, M  And Sano, K | 2002 | Other topic |
| Segmental distraction of the midface in a patient with Crouzon syndrome | Matsumoto, K and Nakanishi, H and Koizumi, Y and Seike, T and Okazaki, M  And Yokozeki, M and Moriyama, K | 2002 | Other topic |
| A comparison of the stability of single-piece and segmental Le Fort I: Maxillary advancements | Arpornmaeklong, P and Heggie, AA and Shand, JM | 2003 | Other topic |
| Midfacial distraction osteogenesis in patients with cleft lip and palate using a segmental osteotomy | Kleinheinz, J and Meyer, U and Joos, U | 2003 | Other topic |
| Midface distraction in an adult patient with Crouzon syndrome | Matsumoto, K and Nakanishi, H and Koutsu, K and Okazaki, M and Moriyama,  K | 2003 | Other topic |
| Two-stage orthognathic treatment of severe class III malocclusion: Report of a case | Iino, M and Ohtani, N and Niitsu, K and Horiuchi, T and Nakamura, Y and  Fukuda, M | 2004 | Other topic |
| [Indications and procedures for segmental dentoalveolar osteotomy: a review of 13 patients.](https://www.google.com/url?q=https://www.google.com/url?q%3Dhttps://pubmed.ncbi.nlm.nih.gov/12592996/%26source%3Dgmail-imap%26ust%3D1685481628000000%26usg%3DAOvVaw0SVyyRoTK9DBI_m0TVwG7b&source=gmail-imap&ust=1694334932000000&usg=AOvVaw1gS4iY0EE2mJOw_bglFJmr) | Yokoo S, Komori T, Watatani S, Shibuya Y, Komurasaki H, Tateishi C.GER | 2002 | Review |
| A prospective study on infectious complications in orthognathic surgery | Spaey, YJE and Bettens, RMA and Mommaerts, MY and Adriaens, J and Van Landuyt, HW and Abeloos, JVS and De Clercq, CAS and Lamoral, PRB and Neyt, LF | 2005 | Other topic |
| [Orthodontic objectives in orthognathic surgery: state of the art today.](https://www.google.com/url?q=https://www.google.com/url?q%3Dhttps://pubmed.ncbi.nlm.nih.gov/16779977/%26source%3Dgmail-imap%26ust%3D1685481628000000%26usg%3DAOvVaw0AJ9Gtj8H8TcD8MCyO3Cj4&source=gmail-imap&ust=1694334932000000&usg=AOvVaw1Qg-WXKeEkMIJUsSincslI) | Sabri R. | 2006 | Other topic |
| Simultaneous maxillary distraction osteogenesis using a twin-track distraction device combined with alveolar bone grafting in cleft patients: Preliminary report of a technique | Suzuki, EY and Watanabe, M and Buranastidporn, B and Baba, Y and Ohyama,  K and Ishii, M | 2006 | Other topic |
| Sagittal and vertical maxillary effects after surgically assisted rapid maxillary expansion (SARME) using Haas and Hyrax expanders | Bretos, J.L.G. and Pereira, M.D. and Gomes, H.C. and Toyama Hino, C. And Ferreira, L.M. | 2007 | Other topic |
| Surgical orthodontic treatment for a patient with unilateral cleft lip and palate and temporomandibular disorder | Kamioka, H. And Ishihara, Y. And Inoue, M. And Takano-Yamamoto, T. | 2007 | Other topic |
| Improvement of vision after a combined midfacial and maxillary distraction with a rigid external distraction device | Tanag, Marvin A. And Takagi, Satoshi and Takashima, Mariko and Nakai,  Kunihiro and Sakai, Yasuo and Yano, Kenji and Takada, Kenji and Hosokawa, Ko | 2007 | Other topic |
| What is Piezosurgery (R)? Two-years experience in craniomaxillofacial surgery | Beziat, J. -L. And Vercellotti, T. And Gleizal, A. | 2007 | Other topic |
| Recombinant human bone morphogenetic protein-2 in absorbable collagen sponge enhances bone healing of tibial osteotomies in dogs | Faria, Maria L. E. And Lu, Yan and Heaney, Kathleen and Uthamanthil,  Rajesh K. And Muir, Peter and Markel, Mark D. | 2007 | Other topic |
| Preparation for Orthognathic Surgery - Arch Width Coordination | Pogrel, M. A. | 2007 | Other topic |
| [Three dimensional distraction osteogenesis of the midface: orthodontic considerations.](https://www.google.com/url?q=https://www.google.com/url?q%3Dhttps://pubmed.ncbi.nlm.nih.gov/22073459/%26source%3Dgmail-imap%26ust%3D1685480936000000%26usg%3DAOvVaw3Z6tV0BgNIqbgVKn1RF5nc&source=gmail-imap&ust=1694334883000000&usg=AOvVaw3JeBnTOXQN-aLsrYuRr0dV) | Aizenbud D, Rachmiel A. | 2008 | Other topic |
| The value of the maxillo-malar osteotomy in the treatment of Crouzon Syndrome with exorbitism | Scafati, Carmine Taglialatela and Aliberti, Ferdinando and Scafati,  Salvatore Taglialatela and Mangone, Giuseppe Michele and Scafati,  Marianna Taglialatela | 2008 | Other topic |
| Good esthetic results after modified maxillomandibular advancement for obstructive sleep apnea syndrome | Matsuo, Akira and Nakai, Takayuki and Toyoda, Jun and Takahashi,  Hidetoshi and Suzuki, Iwao and Chiba, Hiroshige | 2009 | Other topic |
| [Transverse maxillary stability assisted by a transpalatal device: a retrospective pilot study of 9 cases.](https://www.google.com/url?q=https://www.google.com/url?q%3Dhttps://pubmed.ncbi.nlm.nih.gov/19446437/%26source%3Dgmail-imap%26ust%3D1685480936000000%26usg%3DAOvVaw2SHiJKJET4XwpExndLOJlb&source=gmail-imap&ust=1694334883000000&usg=AOvVaw1bJx7Ebk1tyixL-7dTRIt_) | Charezinski M, Balon-Perin A, Deroux E, De Maertelaer V, Glineur R. | 2009 | Other topic |
| [Orthodontic or surgically assisted rapid maxillary expansion.](https://www.google.com/url?q=https://www.google.com/url?q%3Dhttps://pubmed.ncbi.nlm.nih.gov/19590910/%26source%3Dgmail-imap%26ust%3D1685480936000000%26usg%3DAOvVaw38f8ocVd0HjPpg-D6mGtXw&source=gmail-imap&ust=1694334883000000&usg=AOvVaw1W_-AD-4fEPP4n9pmPrmGj) | Chrcanovic BR, Custódio AL. | 2009 | Other topic |
| Stress distribution of surgically assisted tooth-borne rapid maxillary expansion via three different types of osteotomies | Jiang, W. And Wang, X. And Wang, D. | 2009 | Other topic |
| Segmental Le Fort I osteotomy with bone grafting in unilateral severely atrophied maxilla | Pelo, S. And Gasparini, G. And Moro, A. And Boniello, R. And Amoroso, P.  F. | 2009 | Other topic |
| [Influence of the primary cleft palate closure on the future need for orthognathic surgery in unilateral cleft lip and palate patients.](https://www.google.com/url?q=https://www.google.com/url?q%3Dhttps://pubmed.ncbi.nlm.nih.gov/20856058/%26source%3Dgmail-imap%26ust%3D1685481813000000%26usg%3DAOvVaw3QCxK0EeUk9fp7SxNkTWyD&source=gmail-imap&ust=1694334974000000&usg=AOvVaw3eO73ZJNmgV9aWieM3nmxo) | Broome M, Herzog G, Hohlfeld J, de Buys Roessingh A, Jaques B. | 2010 | Other topic |
| Classification of midpalatal suture opening after surgically assisted rapid maxillary expansion using computed tomography | Pereira, M.D. and Prado, G.P.R. and Abramoff, M.M.F. and Aloise, A.C. and Masako Ferreira, L. | 2010 | Other topic |
| Long-term 3D cast model study: Bone-borne vs. Tooth-borne surgically assisted rapid maxillary expansion due to secondary variables | Laudemann, K. And Petruchin, O. And Nafzger, M. And Ballon, A. And Kopp, S. And Sader, R.A. and Landes, C.A.} | 2010 | Other topic |
| [The influence of orthognathic surgery on ventilation during sleep.](https://www.google.com/url?q=https://www.google.com/url?q%3Dhttps://pubmed.ncbi.nlm.nih.gov/21074368/%26source%3Dgmail-imap%26ust%3D1685480936000000%26usg%3DAOvVaw1OG0jmpHtG6YW8h8VlxDaR&source=gmail-imap&ust=1694334883000000&usg=AOvVaw1Tp3biH-W1WfmGt-4gVKTO) | Foltán R, Hoffmannová J, Pavlíková G, Hanzelka T, Klíma K, Horká E, Adámek S, Sedý J. | 2010 | Other topic |
| Intraoperative blood loss in bimaxillary orthognathic surgery with multisegmental Le Fort I osteotomies and additional procedures | Kretschmer, W. B. And Baciut, G. And Bacuit, Mihaela and Zoder, W. And  Wangerin, K. | 2010 | Other topic |
| Rotation Osteotomies In Treatment Of The Post Cleft Deformities Of The Facial Skeleton. | Jagielak, M. J. And Michalski, P. And Jagielak, A. And Socha, M. And  Rogus, P. | 2010 | Other topic |
| [Miniscrew-assisted nonsurgical palatal expansion before orthognathic surgery for a patient with severe mandibular prognathism.](https://www.google.com/url?q=https://www.google.com/url?q%3Dhttps://pubmed.ncbi.nlm.nih.gov/20685540/%26source%3Dgmail-imap%26ust%3D1685480936000000%26usg%3DAOvVaw3p0cY-fxmN7r6PusP4OFBV&source=gmail-imap&ust=1694334883000000&usg=AOvVaw2SgK1k1UZOHQ3BFu3MazAO) | Lee KJ, Park YC, Park JY, Hwang WS. | 2010 | Other topic |
| Soft tissue changes associated with ASO/BSSRO and Le Fort I/BSSRO in Skeletal Class III malocclusion with upper lip protrusion | Kang, Ju-Man and Kim, Yoon-Ji and Park, Je-Uk and Kook, Yoon-Ah | 2010 | Other topic |
| [Orthodontic and orthognathic management of a patient with Apert syndrome: a case report.](https://www.google.com/url?q=https://www.google.com/url?q%3Dhttps://pubmed.ncbi.nlm.nih.gov/20567036/%26source%3Dgmail-imap%26ust%3D1685480936000000%26usg%3DAOvVaw1QOD25lhWutKakm0KA7UxH&source=gmail-imap&ust=1694334883000000&usg=AOvVaw3u67LjyflARutS5-SU7ztc) | Verdonck A, Bertrand J, Carels C, Swinnen S, Schoenaers J. | 2010 | Other topic |
| [Long-term stability of surgical-orthodontic open-bite correction.](https://www.google.com/url?q=https://www.google.com/url?q%3Dhttps://pubmed.ncbi.nlm.nih.gov/20816288/%26source%3Dgmail-imap%26ust%3D1685480936000000%26usg%3DAOvVaw34p2d_Z3rq54Ls3GaIH3lP&source=gmail-imap&ust=1694334883000000&usg=AOvVaw1-bbIatrxGZGFcZe_TMmR3) | Maia FA, Janson G, Barros SE, Maia NG, Chiqueto K, Nakamura AY. | 2010 | Other topic |
| [Sagittal airway changes: rapid palatal expansion versus Le Fort I osteotomy during maxillary protraction.](https://www.google.com/url?q=https://www.google.com/url?q%3Dhttps://pubmed.ncbi.nlm.nih.gov/21464152/%26source%3Dgmail-imap%26ust%3D1685480936000000%26usg%3DAOvVaw0zBt1nzOPuzB0V35LHsPp0&source=gmail-imap&ust=1694334883000000&usg=AOvVaw3XAqXk9r6NPdpt3GinG3ua) | Cakirer B, Kucukkeles N, Nevzatoglu S, Koldas T. | 2011 | Other topic |
| Orthognathic and Osteoperiosteal Flap Augmentation Strategies for Maxillary Dental Implant Reconstruction | Jensen, Ole T. And Ringeman, Jason L. And Cottam, Jared R. And Casap,  Nardy | 2011 | Other topic |
| [The effect of maxillary advancement and impaction on the upper airway after bimaxillary surgery to correct Class III malocclusion.](https://www.google.com/url?q=https://www.google.com/url?q%3Dhttps://pubmed.ncbi.nlm.nih.gov/21435545/%26source%3Dgmail-imap%26ust%3D1685481813000000%26usg%3DAOvVaw1swb5nule1sEeVQ2ww3bhh&source=gmail-imap&ust=1694334974000000&usg=AOvVaw32LK4995J8HU52CTlPpzaJ) | Jakobsone G, Stenvik A, Espeland L. | 2011 | Other topic |
| [Lateral cephalometry changes after SARPE.](https://www.google.com/url?q=https://www.google.com/url?q%3Dhttps://pubmed.ncbi.nlm.nih.gov/21489753/%26source%3Dgmail-imap%26ust%3D1685480936000000%26usg%3DAOvVaw2_u5uWWWy337Eqyp8o3eLc&source=gmail-imap&ust=1694334883000000&usg=AOvVaw2n_EAHvGkmASeZglW2kCBn) | Parhiz A, Schepers S, Lambrichts I, Vrielinck L, Sun Y, Politis C. | 2011 | Other topic |
| Surgical complications of segmental Le Fort I osteotomy | Ho, M. W. And Boyle, M. A. And Cooper, J. C. And Dodd, M. D. And  Richardson, D. | 2011 | Other topic |
| [Determining the osteotomy pattern in surgically assisted rapid maxillary expansion in a unilateral palatal cleft: a finite element model approach.](https://www.google.com/url?q=https://www.google.com/url?q%3Dhttps://pubmed.ncbi.nlm.nih.gov/21299391/%26source%3Dgmail-imap%26ust%3D1685480936000000%26usg%3DAOvVaw0ZtbxBpNCiwZ9Lu0LzScAl&source=gmail-imap&ust=1694334883000000&usg=AOvVaw0WhI53CuOG0BKwhrY7V55J) | Gautam P, Zhao L, Patel P. | 2011 | Other topic |
| [Managing chronic nasal airway obstruction at the time of orthognathic surgery: a twofer.](https://www.google.com/url?q=https://www.google.com/url?q%3Dhttps://pubmed.ncbi.nlm.nih.gov/21353930/%26source%3Dgmail-imap%26ust%3D1685480936000000%26usg%3DAOvVaw0x9LYqiEIZL_BnnnV68HUx&source=gmail-imap&ust=1694334883000000&usg=AOvVaw10dk4-1Y4Pg2sks4NJliZB) | Posnick JC, Agnihotri N. | 2011 | Other topic |
| [Surgery of the transverse dimension.](https://www.google.com/url?q=https://www.google.com/url?q%3Dhttps://pubmed.ncbi.nlm.nih.gov/21627930/%26source%3Dgmail-imap%26ust%3D1685480936000000%26usg%3DAOvVaw2Yy2CQD1TRwoE0OG2jKlfQ&source=gmail-imap&ust=1694334883000000&usg=AOvVaw0DwZmxeKiREDdt_OmscmMI) | Béziat JL. | 2011 | Other topic |
| [Predicted versus executed surgical orthognathic treatment.](https://www.google.com/url?q=https://www.google.com/url?q%3Dhttps://pubmed.ncbi.nlm.nih.gov/23267735/%26source%3Dgmail-imap%26ust%3D1685480936000000%26usg%3DAOvVaw0TODe91EiW6YKaDrMy6dp6&source=gmail-imap&ust=1694334883000000&usg=AOvVaw3NAmgLG3EUsTY6j8vdcrW1) | Falter B, Schepers S, Vrielinck L, Lambrichts I, Politis C. | 2012 | Other topic |
| Simultaneous Le Fort I, II, and III Osteotomies for Correction of Midface Deficiency in Apert Disease | Dai, Jiewen and Wang, Xudong and Yu, Hongbo and Cheng, Jie and Yuan, Hao And Gui, Haijun and Shen, Shunyao and Shen, Guofang | 2012 | Other topic |
| Evaluation of neurosensory alterations via clinical neurosensory test following anterior maxillary osteotomy (Bell technique) | Gulses, A. And Aydintug, Y. S. And Sencimen, M. And Bayar, G. R. And  Acikel, C. H. | 2012 | Other topic |
| [Strategies for surgically assisted rapid maxillary expansion according to the region of transverse maxillary deficiency.](https://www.google.com/url?q=https://www.google.com/url?q%3Dhttps://pubmed.ncbi.nlm.nih.gov/22578567/%26source%3Dgmail-imap%26ust%3D1685481446000000%26usg%3DAOvVaw0VmGBE0HvZKi6C0mCIL4i4&source=gmail-imap&ust=1694334890000000&usg=AOvVaw18TVPrGlwcZpQFbpuhBIG3) | Pereira MD, de Abreu RA, Prado GP, Ferreira LM. | 2012 | Other topic |
| [Surgical-orthodontic treatment for skeletal class II malocclusion with vertical maxillary excess, anterior open bite, and transverse maxillary deficiency.](https://www.google.com/url?q=https://www.google.com/url?q%3Dhttps://pubmed.ncbi.nlm.nih.gov/23172466/%26source%3Dgmail-imap%26ust%3D1685480936000000%26usg%3DAOvVaw09R0hnzLym0cztu2NeD-7L&source=gmail-imap&ust=1694334883000000&usg=AOvVaw1nRITkjRqWz7a48eQR0hiE) | Choi SH, Cha JY, Kang DY, Hwang CJ. | 2012 | Other topic |
| Surgically assisted rapid maxillary expansion. Review of the literature | Andressakis, Dionysios D. | 2012 | Review |
| Strategies for surgically assisted rapid maxillary expansion according to the region of transverse maxillary deficiency | Pereira, M. D. And de Abreu, R. A. M. And Prado, G. P. R. And Ferreira,  L. M. | 2012 | Other topic |
| [Surgical correction of transverse skeletal abnormalities in the maxilla and mandible.](https://www.google.com/url?q=https://www.google.com/url?q%3Dhttps://pubmed.ncbi.nlm.nih.gov/22921345/%26source%3Dgmail-imap%26ust%3D1685480936000000%26usg%3DAOvVaw19uedpTvC1kFSE8vnaDLBj&source=gmail-imap&ust=1694334883000000&usg=AOvVaw29ndBTEMBQHE96w_QFn8In) | Bouletreau P, Paulus C. | 2012 | Other topic |
| [Glossectomy as an adjunct to correct an open-bite malocclusion with shortened maxillary central incisor roots.](https://www.google.com/url?q=https://www.google.com/url?q%3Dhttps://pubmed.ncbi.nlm.nih.gov/23810054/%26source%3Dgmail-imap%26ust%3D1685481813000000%26usg%3DAOvVaw1br1tbSJ6kNKGkrZERzecK&source=gmail-imap&ust=1694334974000000&usg=AOvVaw22ecTp_cq7d9XOYERW_r_1) | Tanaka OM, Guariza-Filho O, Carlini JL, Oliveira DD, Pithon MM, Camargo ES. | 2013 | Other topic |
| Effects of surgically assisted rapid palatal expansion with and without pterygomaxillary disjunction on dental and skeletal structures: A retrospective review | Kilic, E. And Kilic, B. And Kurt, G. And Sakin, C. And Alkan, A. | 2013 | Other topic |
| [Arch coordination does not affect the stability in class III orthognathic surgery patients.](https://www.google.com/url?q=https://www.google.com/url?q%3Dhttps://pubmed.ncbi.nlm.nih.gov/24220471/%26source%3Dgmail-imap%26ust%3D1685481813000000%26usg%3DAOvVaw0MBsgVSUBXzbEzDvg1qqb5&source=gmail-imap&ust=1694334974000000&usg=AOvVaw2QKPXcDHaRDifpe4FTcWdT) | Hong SO, Ryu DM, Lee DW, Jung JH. | 2013 | Other topic |
| [Surgical-orthodontic correction of a skeletal Class III malocclusion with severe maxillary constriction.](https://www.google.com/url?q=https://www.google.com/url?q%3Dhttps://pubmed.ncbi.nlm.nih.gov/24225165/%26source%3Dgmail-imap%26ust%3D1685480936000000%26usg%3DAOvVaw3qZUP2ipqqfIX9G4j0GvRQ&source=gmail-imap&ust=1694334883000000&usg=AOvVaw3ANPo2E5nv7M0wBDRMq-Zv) | Wang Y, Liu M, Zhou L, Hu J. | 2013 | Other topic |
| [Multidisciplinary management of a child with severe open bite and amelogenesis imperfecta.](https://www.google.com/url?q=https://www.google.com/url?q%3Dhttps://pubmed.ncbi.nlm.nih.gov/23811667/%26source%3Dgmail-imap%26ust%3D1685480936000000%26usg%3DAOvVaw0QaWhzArgKctyAS3GU7kLO&source=gmail-imap&ust=1694334883000000&usg=AOvVaw3zIp2QbVPhirGvTMS7DOLQ) | Millet C, Duprez JP. | 2013 | Other topic |
| [Analysis of incision effects on upper lip height and thickness after maxillary surgically assisted expansion: a randomized clinical trial.](https://www.google.com/url?q=https://www.google.com/url?q%3Dhttps://pubmed.ncbi.nlm.nih.gov/23890785/%26source%3Dgmail-imap%26ust%3D1685480936000000%26usg%3DAOvVaw2n8k8ww3o49bfJRGUGlq9s&source=gmail-imap&ust=1694334883000000&usg=AOvVaw0xtNarfAhIf-mpyelxtxNO) | Antonini F, da Costa DJ, de Moares RS, Rebellato NL, Klüppel LE, Parente EV. | 2013 | Other topic |
| [Finite element analysis of stress distribution in anchor teeth in surgically assisted rapid palatal expansion.](https://www.google.com/url?q=https://www.google.com/url?q%3Dhttps://pubmed.ncbi.nlm.nih.gov/23684813/%26source%3Dgmail-imap%26ust%3D1685480936000000%26usg%3DAOvVaw2UGO-ynTvj7meSAvGSRS2m&source=gmail-imap&ust=1694334883000000&usg=AOvVaw2gGtXqjJbyBL5F3IkUT6sl) | De Assis DS, Xavier TA, Noritomi PY, Gonçales AG, Ferreira O Jr, de Carvalho PC, Gonçales ES. | 2013 | Other topic |
| [Successful orthodontic-surgical treatment: aiming for esthetics and function. Analysis of some clinical cases.](https://www.google.com/url?q=https://www.google.com/url?q%3Dhttps://pubmed.ncbi.nlm.nih.gov/24183536/%26source%3Dgmail-imap%26ust%3D1685480936000000%26usg%3DAOvVaw2X_-6cqm8Yo1hZaSFaKUG6&source=gmail-imap&ust=1694334883000000&usg=AOvVaw0BQwr_udfWVSKnWirg4ovu) | Frapier L, Garcia C, Pic E, Morant F, Belguendouz S, Gauthier A, Raynal P. | 2013 | Other topic |
| [Surgically assisted rapid maxillary expansion: feasibility of not releasing the nasal septum.](https://www.google.com/url?q=https://www.google.com/url?q%3Dhttps://pubmed.ncbi.nlm.nih.gov/23092854/%26source%3Dgmail-imap%26ust%3D1685481628000000%26usg%3DAOvVaw21J6VyTamVoHxDNCI7VCWI&source=gmail-imap&ust=1694334932000000&usg=AOvVaw2eRyStnfs9UrMT123nx3TG) | Reinbacher KE, Wallner J, Pau M, Feichtinger M, Kärcher H, Quehenberger F, Zemann W. | 2013 | Other topic |
| Finite Element Analysis of Bone Stress After SARPE | Ferreira Rubim de Assis, Diogo Souza and Xavier, Tathy Aparecida and  Noritomi, Pedro Yoshito and Goncales, Eduardo Sanches | 2014 | Other topic |
| Surgically assisted rapid maxillary expansion. An evaluation of different surgical techniques and their effect on maxillary dentoskeletal complex based on cone-beam computed tomography. Preliminary report | Sygouros, A. And Motro, M. And Ugurlu, F. And Acar, A. | 2014 | Preliminary study |
| Piezosurgery for surgically assisted rapid maxillary expansion under local anesthesia | Sun, H. And Li, B. And Liu, Z. And Wang, X. | 2014 | Other topic |
| [Treacher Collins syndrome: a case study.](https://www.google.com/url?q=https://www.google.com/url?q%3Dhttps://pubmed.ncbi.nlm.nih.gov/25439217/%26source%3Dgmail-imap%26ust%3D1685480936000000%26usg%3DAOvVaw3Ayr47p7-yx0-xMhY7Mx4a&source=gmail-imap&ust=1694334883000000&usg=AOvVaw3s2KqfmiDShgTrBi6zuULU) | Chung JY, Cangialosi TJ, Eisig SB. | 2014 | Other topic |
| [Adult patient with hemifacial microsomia treated with combined orthodontics and distraction osteogenesis.](https://www.google.com/url?q=https://www.google.com/url?q%3Dhttps://pubmed.ncbi.nlm.nih.gov/24373657/%26source%3Dgmail-imap%26ust%3D1685481628000000%26usg%3DAOvVaw11q8DgrrxX3oSkrOiGRjJS&source=gmail-imap&ust=1694334932000000&usg=AOvVaw3KZ-z42W84ypjIGWa3xbCu) | Choi SH, Kang DY, Hwang CJ. | 2014 | Other topic |
| [Severe skeletal Class III malocclusion treated with 2-stage orthognathic surgery with a mandibular step osteotomy.](https://www.google.com/url?q=https://www.google.com/url?q%3Dhttps://pubmed.ncbi.nlm.nih.gov/24680021/%26source%3Dgmail-imap%26ust%3D1685481813000000%26usg%3DAOvVaw1B050ayhud74q6DuPn6gpq&source=gmail-imap&ust=1694334974000000&usg=AOvVaw0mdEpnCNCKTrvP5wzgpg74) | Choi SH, Kang DY, Kim YH, Hwang CJ. | 2014 | Other topic |
| [Maxillary orthognathic surgery.](https://www.google.com/url?q=https://www.google.com/url?q%3Dhttps://pubmed.ncbi.nlm.nih.gov/25199863/%26source%3Dgmail-imap%26ust%3D1685480936000000%26usg%3DAOvVaw31Qu-BdUWIxU3I6I0IS05p&source=gmail-imap&ust=1694334883000000&usg=AOvVaw0Dmn7_hyvEqb3QKtknLxTE) | [Richard E Bauer 3rd](https://pubmed.ncbi.nlm.nih.gov/?term=Bauer+RE+3rd&cauthor_id=25199863) , [Mark W Ochs](https://pubmed.ncbi.nlm.nih.gov/?term=Ochs+MW&cauthor_id=25199863) | 2014 | Review |
| [Soft tissue response and facial symmetry after orthognathic surgery.](https://www.google.com/url?q=https://www.google.com/url?q%3Dhttps://pubmed.ncbi.nlm.nih.gov/24529350/%26source%3Dgmail-imap%26ust%3D1685480936000000%26usg%3DAOvVaw32Z8drmHw1w2Kic3TNc9nn&source=gmail-imap&ust=1694334883000000&usg=AOvVaw0lUvTMkFsen7IOj8jVw7Sv) | Wermker K, Kleinheinz J, Jung S, Dirksen D. | 2014 | Other topic |
| Recovery of maxillary tooth sensibility after Le Fort I osteotomy | Bonnot, P. And Salles, F. And Cheynet, F. And Blanc, J. L. And Ricbourg,  B. And Meyer, C. | 2014 | Other topic |
| [Interdisciplinary treatment of a patient with bilateral cleft lip and palate and congenitally missing and transposed teeth.](https://www.google.com/url?q=https://www.google.com/url?q%3Dhttps://pubmed.ncbi.nlm.nih.gov/24582029/%26source%3Dgmail-imap%26ust%3D1685480936000000%26usg%3DAOvVaw0vpnA7QbxU44iBQ79hf3SN&source=gmail-imap&ust=1694334883000000&usg=AOvVaw2n56W6ZWvzhZ2QdEFJ4tGS) | Germec-Cakan D, Canter HI, Cakan U, Demir B. | 2014 | Other topic |
| Single Versus Segmental Maxillary Osteotomies and Long-Term Stability in Unilateral Cleft Lip and Palate Related Malocclusion | Watts, Guy D. And Antonarakis, Gregory S. And Forrest, Christopher R.  And Tompson, Bryan D. And Phillips, John H. | 2014 | Other topic |
| [Long-term orthodontic and surgical treatment and stability of a patient with Beckwith-Wiedemann syndrome.](https://www.google.com/url?q=https://www.google.com/url?q%3Dhttps://pubmed.ncbi.nlm.nih.gov/24785932/%26source%3Dgmail-imap%26ust%3D1685480936000000%26usg%3DAOvVaw0xvSz7URn5PYLtZdzTq0G1&source=gmail-imap&ust=1694334883000000&usg=AOvVaw1PQtfTrp_-6CAqcmGFXBco) | Hikita R, Kobayashi Y, Tsuji M, Kawamoto T, Moriyama K. | 2014 | Other topic |
| [One-step surgery and correction of the transverse dimension of the maxilla using the lingual technique: an original assisted transverse surgery method.](https://www.google.com/url?q=https://www.google.com/url?q%3Dhttps://pubmed.ncbi.nlm.nih.gov/25108522/%26source%3Dgmail-imap%26ust%3D1685480936000000%26usg%3DAOvVaw3L6HHEYKvf6iUaZ0cdwwG9&source=gmail-imap&ust=1694334883000000&usg=AOvVaw3-GMZ8Qs4GAIagi3Qrr0HR) | Galletti C, Deffrennes D. | 2014 | Other topic |
| [Oral focal mucinosis associated with surgically assisted rapid maxillary expansion.](https://www.google.com/url?q=https://www.google.com/url?q%3Dhttps://pubmed.ncbi.nlm.nih.gov/24703292/%26source%3Dgmail-imap%26ust%3D1685480936000000%26usg%3DAOvVaw3G-_GtUFqAZvomnm1k7tUL&source=gmail-imap&ust=1694334883000000&usg=AOvVaw1DHX7AyOUJc8s4Q48BHYDo) | Neto JR, Sendyk M, Uchida LM, Nunes FD, de Paiva JB. | 2014 | Other topic |
| [[Surgically assisted rapid maxillary expansion. An evaluation of different surgical techniques and their effect on maxillary dentoskeletal complex based on cone-beam computed tomography. Preliminary report].](https://www.google.com/url?q=https://www.google.com/url?q%3Dhttps://pubmed.ncbi.nlm.nih.gov/24923217/%26source%3Dgmail-imap%26ust%3D1685481446000000%26usg%3DAOvVaw3_FVQsfC5WRPGZml0MsZ0Z&source=gmail-imap&ust=1694334890000000&usg=AOvVaw1fyta_Bf6ERJ_y36fe-Pcr) | Sygouros A, Motro M, Ugurlu F, Acar A. | 2014 | Preliminary study |
| Dimensions of Velopharyngeal Space following Maxillary Advancement with Le Fort I Osteotomy Compared to Zisser Segmental Osteotomy: A Cephalometric Study | Karabekmez, Furkan Erol and Kleinheinz, Johannes and Jung, Susanne | 2015 | Other topic |
| [A Preliminary Three-Dimensional Analysis of Nasal Aesthetics Following Le Fort I Advancement in Patients With Cleft Lip and Palate.](https://www.google.com/url?q=https://www.google.com/url?q%3Dhttps://pubmed.ncbi.nlm.nih.gov/26468849/%26source%3Dgmail-imap%26ust%3D1685481813000000%26usg%3DAOvVaw2S-3bzAC6ib6WbnnFlzalU&source=gmail-imap&ust=1694334974000000&usg=AOvVaw0afWifOE2778JtMxW8BEHr) | Davidson E, Kumar AR | 2015 | Other topic |
| [Long-term skeletal and dental stability after orthognathic surgery of the maxillo-mandibular complex in Class II patients with transverse discrepancies.](https://www.google.com/url?q=https://www.google.com/url?q%3Dhttps://pubmed.ncbi.nlm.nih.gov/26293193/%26source%3Dgmail-imap%26ust%3D1685480936000000%26usg%3DAOvVaw1A7gkdzTYL89rEgsQbcsLI&source=gmail-imap&ust=1694334883000000&usg=AOvVaw1awY4p0ziZXhtFfJF5z1ex) | Brandtner C, Hachleitner J, Rippel C, Krenkel C, Gaggl A. | 2015 | Other topic |
| [Comprehensive treatment approach for bilateral cleft lip and palate in an adult with premaxillary osteotomy, tooth autotransplantation, and 2-jaw surgery.](https://www.google.com/url?q=https://www.google.com/url?q%3Dhttps://pubmed.ncbi.nlm.nih.gov/25533078/%26source%3Dgmail-imap%26ust%3D1685480936000000%26usg%3DAOvVaw23YhGT6kKNjEJWg2hqGnxp&source=gmail-imap&ust=1694334883000000&usg=AOvVaw1XFgWxMW7msEIb2k00cuRJ) | Kokai S, Fukuyama E, Sato Y, Hsu JC, Takahashi Y, Harada K, Ono T | 2015 | Other topic |
| [Combined surgical-orthodontic treatment: how did it evolve and what are the best practices now?](https://www.google.com/url?q=https://www.google.com/url?q%3Dhttps://pubmed.ncbi.nlm.nih.gov/25925650/%26source%3Dgmail-imap%26ust%3D1685480936000000%26usg%3DAOvVaw1xYflJLCGwj4h6ldQvsuPb&source=gmail-imap&ust=1694334883000000&usg=AOvVaw0azGozr4VfPtOMhkS8LRnt) | Proffit WR, White RP Jr. | 2015 | Other topic |
| [Surgery Versus Nonsurgery Option for Scissors Bite Treatment.](https://www.google.com/url?q=https://www.google.com/url?q%3Dhttps://pubmed.ncbi.nlm.nih.gov/26594987/%26source%3Dgmail-imap%26ust%3D1685481813000000%26usg%3DAOvVaw1fRuRC-yYDk7DDBYMnZ9Wv&source=gmail-imap&ust=1694334974000000&usg=AOvVaw08wFHMttxtE18XJ4u8fTGy) | Kim KA, Yu JJ, Chen Y, Kim SJ, Kim SH, Nelson G. | 2015 | Other topic |
| Modified maxillomandibular advancement for obstructive sleep apnoea: Towards a better outcome for Asians | Liao, Y-F. And Chiu, Y-T. And Lin, C-H. And Chen, Y-A. And Chen, N-H.  And Chen, Y-R. | 2015 | Other topic |
| Interdisciplinary Treatment for an Adult Patient With Anterior Open Bite, Severe Periodontitis, and Intellectual Disability | Kang, Da-Young and Choi, Sung-Hwan and Jung, Young-Soo and Hwang,  Chung-Ju | 2015 | Other topic |
| 45 Years of Simultaneous Le Fort III and Le Fort I Osteotomies: A Systematic Literature Review | Brown, Matthew S. And Okada, Haruko and Valiathan, Manish and Lakin, Gregory E. | 2015 | SR |
| Modified SARME (surgically assisted rapid maxillary expansion) in conjunction with orthodontic treatment- a case report | Dahiya, S. And Chitra, P. And Rao, S.S. and Bindra, S. | 2015 | Other topic |
| Salvage rapid maxillary expansion for the relapse of maxillary transverse expansion after Le Fort I with parasagittal osteotomy | Lee, Hyun-Woo and Kim, Su-Jung and Kwon, Yong-Dae | 2015 | Other topic |
| Surgically assisted rapid palatomaxillary expansion with or without pterygomaxillary disjunction: a systematic review and meta-analysis. | Sangsari, A. H., Sadr-Eshkevari, P., Al-Dam, A., Friedrich, R. E., Freymiller, E., & Rashad, A. | 2016 | SR |
| Three-dimensional analysis of maxillary stability after Le Fort I osteotomy using hydroxyapatite/poly-L-lactide plate | Park, Jung-Hyun and Kim, Minkyu and Kim, Sang Yoon and Jung, Hwi-Dong  And Jung, Young-Soo | 2016 | Other topic |
| Unilateral Le Fort I Osteotomy for Rehabilitating the Large Alveolar Cleft and Vertical Malocclusion With the Distraction Technique | Baykul, Timucin and Aydin, Mustafa Asim and Findik, Yavuz and Esenlik,  Elcin | 2016 | Other topic |
| Segmental Maxillary Osteotomies in Conjunction With Bimaxillary Orthognathic Surgery: Indications - Safety - Outcome | Posnick, Jeffrey C. And Adachie, Anayo and Choi, Elbert | 2016 | Other topic |
| Three-dimensional nasolabial morphologic alterations following Le Fort I | Desesa, C.R. and Metzler, P. And Sawh-Martinez, R. And Steinbacher, D.M. | 2016 | Other topic |
| Biomechanical interactions of different mini-plate fixations and maxilla advancements in the Le Fort I Osteotomy: a finite element analysis | Huang, Shao-Fu and Lo, Lun-Jou and Lin, Chun-Li | 2016 | Other topic |
| Maxillary changes with bone-borne surgically assisted rapid palatal  Expansion: A prospective study | Asscherickx, Karlien and Govaerts, Elke and Aerts, Johan and Vande  Vannet, Bart | 2016 | Other topic |
| Prevalence and risk factors of tooth discolouration after orthognathic surgery: a retrospective study of 1455 patients | Lee, U. -L. And Lee, E. -J. And Seo, H. -Y. And Han, S. -H. And Choi, W.  -C. And Choi, Y. -J. | 2016 | Other topic |
| Massive Middle Cerebral Artery Infarction After Surgically Assisted Rapid Palatal Expansion: A Case Report | Kufta, Kenneth and Melean, Luis Perez and Grady, M. Sean and Panchal,  Neeraj | 2017 | Other topic |
| Application of 4-Meta Adhesive Resin to the Occlusal Surface During Surgery Facilitates Postoperative Occlusal Management | Noriaki, Aoki and Junichi, Baba and Toshinori, Iwai and Iwai, Tohnai | 2017 | Other topic |
| [Simulation of three surgical techniques combined with two different bone-borne forces for surgically assisted rapid palatal expansion of the maxillofacial complex: a finite element analysis](https://www.scopus.com/record/display.uri?eid=2-s2.0-85020472316&origin=resultslist) | Möhlhenrich, S.C., Modabber, A., Kniha, K., Peters, F., Steiner, T., Hölzle, F., Fritz, U., Raith, S. | 2017 | Other topic |
| Does intravenous tranexamic acid reduce blood loss during surgically assisted rapid palatal expansion? | Akbas, Emine and Cebi, Zerrin and Cansiz, Erol and Isler, Sabri Cemil  And Cakarer, Sirmahan | 2017 | Other topic |
| [Computer-Assisted Virtual Planning for Surgical Guide Manufacturing and Internal Distractor Adaptation in the Management of Midface Hypoplasia in Cleft Patients.](https://www.google.com/url?q=https://www.google.com/url?q%3Dhttps://pubmed.ncbi.nlm.nih.gov/27223625/%26source%3Dgmail-imap%26ust%3D1685480936000000%26usg%3DAOvVaw1_8IeOXmXHJCXxOonVJmWm&source=gmail-imap&ust=1694334883000000&usg=AOvVaw1Fe0tUtJ285MqVTihQmAE9) | Scolozzi P, Herzog G. | 2017 | Other topic |
| [Maxillary Expansion and Mandibular Setback Surgery With and Without Mandibular Anterior Segment Osteotomy to Correct Mandibular Prognathism With Obstructive Sleep Apnea.](https://www.google.com/url?q=https://www.google.com/url?q%3Dhttps://pubmed.ncbi.nlm.nih.gov/28060088/%26source%3Dgmail-imap%26ust%3D1685481628000000%26usg%3DAOvVaw2d8cqr8AH2DSRzSJvrmNNr&source=gmail-imap&ust=1694334932000000&usg=AOvVaw032xlyBI85V2Gh5ek-y08r) | Han JJ, Hong DH, Hwang SJ. | 2017 | Other topic |
| Stability and surgical complications in segmental Le Fort I osteotomy: a Systematic review | Haas Junior, O. L. And Guijarro-Martinez, R. And de Sousa Gil, A. P. And  Da Silva Meirelles, L. And de Oliveira, R. B. And Hernandez-Alfaro, F. | 2017 | SR |
| Midfacial Changes Through Anterior Maxillary Distraction Osteogenesis in Patients With Cleft Lip and Palate | Kanzaki, Hiroyuki and Imai, Yoshimichi and Nakajo, Tetsu and Daimaruya, Takayoshi and Sato, Akimitsu and Tachi, Masahiro and Nunomura, Youhei And Itagaki, Yusuke and Nishimura, Kazuaki and Kochi, Shoko and Igarashi, Kaoru | 2017 | Other topic |
| [Modified technique used for sagittal splitting of the mandible.](https://www.google.com/url?q=https://www.google.com/url?q%3Dhttps://pubmed.ncbi.nlm.nih.gov/28391077/%26source%3Dgmail-imap%26ust%3D1685481813000000%26usg%3DAOvVaw3VbDjxRLxD8qMpAUQ6vXtQ&source=gmail-imap&ust=1694334974000000&usg=AOvVaw1uP6kF5XUPZ7-oo3rP-nzW) | Loncle T, Bontemps P, Bénaicha M. | 2017 | Other topic |
| [Maxillary Interdental Osteotomies Have Low Morbidity for Alveolar Crestal Bone and Adjacent Teeth: A Cone Beam Computed Tomography-Based Study.](https://www.google.com/url?q=https://www.google.com/url?q%3Dhttps://pubmed.ncbi.nlm.nih.gov/29544755/%26source%3Dgmail-imap%26ust%3D1685481813000000%26usg%3DAOvVaw3ujJoLXoOpLgskumQZ5cNi&source=gmail-imap&ust=1694334974000000&usg=AOvVaw1kE0Bg2FelZ13CyCJ5HIjm) | Rodrigues DB, Campos PSF, Wolford LM, Ignácio J, Gonçalves JR. | 2018 | Other topic |
| Evaluation of stress by finite element analysis of the midface and skull base at the time of midpalatal osteotomy in models with or without pterygomaxillary dysjunction | Esen, A. And Soganci, E. And Dolanmaz, E. And Dolanmaz, D. | 2018 | Other topic |
| [Rapid Anterior Segmental Maxillary Retraction by Compression Osteogenesis.](https://www.google.com/url?q=https://www.google.com/url?q%3Dhttps://pubmed.ncbi.nlm.nih.gov/29194268/%26source%3Dgmail-imap%26ust%3D1685481628000000%26usg%3DAOvVaw0U2_8xpCSDhiy3D-gElrRa&source=gmail-imap&ust=1694334932000000&usg=AOvVaw3hjStaDNHto80ssrTc8N78) | Hellal US, Fayed N, Elsharkawy R, Abdelrahmen M. | 2018 | Other topic |
| [Intra- and Postoperative Complications of Le Fort I Maxillary Osteotomy.](https://www.google.com/url?q=https://www.google.com/url?q%3Dhttps://pubmed.ncbi.nlm.nih.gov/30277955/%26source%3Dgmail-imap%26ust%3D1685481813000000%26usg%3DAOvVaw1mC5zYSuCcS_GRz3OFeKeM&source=gmail-imap&ust=1694334974000000&usg=AOvVaw3NxWfbz9R0QgFJ-U8DAnd2) | Eshghpour M, Mianbandi V, Samieirad S. | 2018 | Other topic |
| Laser-Assisted Indocyanine Green Imaging for Assessment of Perioperative Maxillary Perfusion During Le Fort I Osteotomy: A Pilot Study | Han, Michael D. And Miloro, Michael and Markiewicz, Michael R. | 2018 | Other topic |
| Annual review of selected scientific literature: A report of the Committee on Scientific Investigation of the American Academy of Restorative Dentistry. | Donovan, T. E., Marzola, R., Murphy, K. R., Cagna, D. R., Eichmiller, F., mckee, J. R., ... & Troeltzsch, M. | 2018 | Review |
| Segmental Multiple-Jaw Surgery without Orthodontia: Clear Aligners Alone | Kankam, Hadyn K. N. And Gupta, Himank and Sawh-Martinez, Rajendra and  Steinbacher, Derek M. | 2018 | Other topic |
| Complications from Surgically Assisted Rapid Maxillary Expansion with HAAS and HYRAX Expanders | Pereira, M.D. and Koga, A.F. and Prado, G.P.R. and Ferreira, L.M. | 2018 | Other topic |
| Reaching the vertical versus horizontal target position in multi-segmental Le Fort I osteotomy is more difficult, but yields comparably stable results to one-segment osteotomy | Meewis, J. And Govaerts, D. And Falter, B. And Grisar, K. And Shaheen, E. And Van de Vyvere, G. And Politis, C. | 2018 | Other topic |
| Dynamic analysis of maxillary perfusion during Le Fort I osteotomy using indocyanine green | Salman, S. And Fattahi, T. And Fernandes, R. And Steinberg, B. | 2018 | Other topic |
| [Adjustable selective maxillary expansion combined with one-stage maxillomandibular surgery: A prospective study of osseous widening in fifty-five consecutive patients.](https://www.google.com/url?q=https://www.google.com/url?q%3Dhttps://pubmed.ncbi.nlm.nih.gov/30001885/%26source%3Dgmail-imap%26ust%3D1685480936000000%26usg%3DAOvVaw1HogbVnnC8RAUKuu1WBo7g&source=gmail-imap&ust=1694334883000000&usg=AOvVaw252Uq5nhph_8wdr9fjsgCO) | Leyder P, Altounian G, Quilichini J. | 2018 | Other topic |
| Surgically assisted rapid maxillary expansion with bone-borne versus tooth-borne distraction appliances-a systematic review | Blaehr, TL, Mommaerts, MY, Kjellerup, AD, Starch-Jensen, T | 2019 | SR |
| Does surgically assisted rapid maxillary expansion associated with pterygomaxillary disjunction result in changes in mandibular position? | Carvalho, F. S. R., Soares, E. C. S., Barbosa, D. A. F., de Araújo Mouta, A. E., Bezerra, T. M. M., Ribeiro, T. R., & Costa, F. W. G. | 2019 | Other topic |
| Interproximal bone in maxillary anterior teeth in subjects with Class  III facial deformity: Are there options for segmental maxillary osteotomy in ``surgery first{''}? | Brito, L. And Olate, S. And Villa, J. And Navarro, P. And Haidar, Z. S.  And de Moraes, M. | 2019 | Other topic |
| [Tunnel technique through three vertical buccal incisions to reduce nasolabial changes due to surgically assisted rapid palatal expansion](https://www.scopus.com/record/display.uri?eid=2-s2.0-85062978918&origin=resultslist) | Santagata, M., Corvo, G., Chagas-Júnior, O.L., Tartaro, G. | 2019 | Other topic |
| [Maintenance of Segmental Maxillary Expansion: The Use of Custom, Virtually Designed, and Manufactured Palatal Appliances Without the Use of an Occlusal Splint.](https://www.google.com/url?q=https://www.google.com/url?q%3Dhttps://pubmed.ncbi.nlm.nih.gov/31002787/%26source%3Dgmail-imap%26ust%3D1685480936000000%26usg%3DAOvVaw3emeoBueocRTLk_ASo-LGa&source=gmail-imap&ust=1694334883000000&usg=AOvVaw38AYrKkwSOMrtlnJHOvlDZ) | Ismail M, Wessel J, Farrell B. | 2019 | Other topic |
| Modified technique of U-shaped segmental osteotomy of the palate in the correction of vertical and transverse maxillary deformities | Li, W. And Zhang, L. And Xi, M. And Wang, T. | 2019 | Other topic |
| Excessive gingival display treated with 2-piece segmental Le Fort I osteotomy | Hichijo, Natsuko and Furutani, Masahiro and Kuroda, Shingo and Tanaka, Eiji | 2019 | Other topic |
| Cone beam computed tomography evaluation of tooth injury after segmental Le Fort I osteotomy | Hartlev, J. And Pedersen, T. Klit and Norholt, S. E. | 2019 | Other topic |
| [Comparison of the Planned Versus Actual Jaw Movement Using Splint-Based Virtual Surgical Planning: How Close Are We at Achieving the Planned Outcomes?](https://www.google.com/url?q=https://www.google.com/url?q%3Dhttps://pubmed.ncbi.nlm.nih.gov/30959009/%26source%3Dgmail-imap%26ust%3D1685481813000000%26usg%3DAOvVaw0bYkK8f6gnrQrQkvVbVoJh&source=gmail-imap&ust=1694334974000000&usg=AOvVaw0HI3Evgk6Xxf0giX6DUy3u) | Tankersley AC, Nimmich MC, Battan A, Griggs JA, Caloss R. | 2019 | Other topic |
| [Surgical/Orthodontic Correction of Transverse Maxillary Discrepancies.](https://www.google.com/url?q=https://www.google.com/url?q%3Dhttps://pubmed.ncbi.nlm.nih.gov/31699580/%26source%3Dgmail-imap%26ust%3D1685481628000000%26usg%3DAOvVaw3lNHMuRPjZeWlYtIyeGOwW&source=gmail-imap&ust=1694334932000000&usg=AOvVaw0ki4U93zIyUrkC0L1JVGUa) | Reyneke JP, Conley RS. | 2020 | Other topic |
| Surgically assisted rapid maxillary expansion: a systematic review of complications | Carvalho, P. H. A., Moura, L. B., Trento, G. S., Holzinger, D., Gabrielli, M. A. C., Gabrielli, M. F. R., & Pereira Filho, V. A. | 2020 | SR |
| Pterygomaxillary Disjunction and its Influence on the Result of Surgically Assisted Maxillary Expansion: A Systematic Review and Meta-analysis. | Da Silva Mesquita, B., do Egito Vasconcelos, B. C., de Moraes, S. L. D., Lemos, C. A. A., de Luna Gomes, J. M., Pellizzer, E. P., & de Souza Andrade, E. S. | 2020 | SR |
| Orthognathic surgery with two-segment le fort i and sagittal split ramus osteotomies of open bite deformity in an amelogenesis imperfecta patient viavirtual planning: A case report | Ertas, U. And Ataol, M. And Kiki, A. And Ugurlu, M. | 2020 | Other topic |
| [Skeletal and dental effects of surgically assisted rapid palatal expansion: A systematic review of randomized controlled trials](https://www.scopus.com/record/display.uri?eid=2-s2.0-85090911855&origin=resultslist) | Bortolotti, F., Solidoro, L., Bartolucci, M.L., Incerti Parenti, S., Paganelli, C., Alessandri-Bonetti, G. | 2020 | SR |
| [A comparison of tooth-borne and bone-anchored expansion devices in SARME](https://www.scopus.com/record/display.uri?eid=2-s2.0-85083420232&origin=resultslist) | Barone, T.R., Cahali, M.B., Vasconcelos, C., Barone, J.R. | 2020 | Other topic |
| Can palatal splint improve stability of segmental Le Fort I osteotomies? | Parizotto, Julianna Oliveira Lima and Borsato, Karina Tostes and Peixoto, Adriano Porto and Bianchi, Jonas and Cassano, Daniel Serra and Goncalves, Joao Roberto | 2020 | Other topic |
| Relapse rate after surgical treatment of maxillary hypoplasia in non-growing cleft patients: a systematic review and meta-analysis | Jiang, L. And Zheng, Y. And Li, N. And Chen, X. And Lu, Z. And Tong, H. And Yin, N. And Song, T | 2020 | Other topic |
| Evaluation of Tissue Tensions in Segmental Maxillary Osteotomies by Finite Element Analysis | Sommerfeld, Ricardo and Bergamaschi, Isabela P. And Scariot, Rafaela and Da Costa, Delson J. | 2020 | Other topic |
| Orthognathic surgery treatment injuries reported to the Danish Patient Compensation Association: A 25-year retrospective observational study | Hillerup, S. | 2020 | Other topic |
| Craniofacial Deformities in Patients With Beta-Thalassemia: Orthodontic Versus Surgical Correction-A Systematic Review | Einy, Shmuel and Ben-Barak, Ayelet and Kridin, Khalaf and Aizenbud, Dror | 2020 | SR |
| Technical Modifications Specific to the Cleft Le Fort I Osteotomy | Susarla, Srinivas M. And Ettinger, Russell and Preston, Kathryn and Kapadia, Hitesh and Egbert, Mark A. | 2020 | Other topic |
| Study of soft tissue changes in the upper lip and nose after backward movement of the maxilla in orthognathic surgery | Seon, Suyun and Lee, Hyun-Woo and Jeong, Bong-Jin and Lee, Baek-Soo and Kwon, Yong-Dae and Ohe, Joo-Young | 2020 | Other topic |
| Two-point nasomaxillary fixation of the Le Fort I osteotomy: assessment  Of stability at one year postoperative | Susarla, S. M. And Ettinger, R. And Preston, K. And Kapadia, H. And Egbert, M. A. | 2020 | Other topic |
| Is alteration of the occlusal plane stable with isolated nasomaxillary fixation of the Le Fort I osteotomy? | Susarla, S. M. And Ettinger, R. E. And Preston, K. And Kapadia, H. And Egbert, M. A. | 2020 | Other topic |
| Resorbable Versus Titanium Fixation of Le Fort I Osteotomy | Passeri, Luis Augusto and Bento, Adriano Mesquita and Vanni, Tazio | 2020 | Other topic |
| Microvascular Reconstruction of Total Maxillary Avascular Necrosis as a Complication of Routine Orthognathic Surgery | Ettinger, Kyle S. And Nathan, John and Guerrero, Lidia M. And Salinas, Thomas J. And Arce, Kevin | 2020 | Other topic |
| [Patient satisfaction after orthognathic surgery: a 3 year follow-up of 60 high-angle Class II individuals.](https://www.google.com/url?q=https://www.google.com/url?q%3Dhttps://pubmed.ncbi.nlm.nih.gov/32562420/%26source%3Dgmail-imap%26ust%3D1685481813000000%26usg%3DAOvVaw06AjU60lqqPQUhDR7Fos_k&source=gmail-imap&ust=1694334974000000&usg=AOvVaw3DNUE1BUOEOQjydsUh4NBi) | Torgersbråten N, Stenvik A, Espeland L. | 2021 | Other topic |
| Maxillary repositioning using a CAD/CAM wafer and an intraoperative Navigation system for bimaxillary orthognathic surgery using segmental Le Fort I osteotomy: A pilot study | Tanaka, Motohiro and Sato, Hitoshi and Inada, Takanobu and Yaso, Atsutosi and Ogura, Hiroshi and Shirota, Tatsuo | 2021 | Other topic |
| [Virtual Analysis of Segmental Bimaxillary Surgery: A Validation Study.](https://www.google.com/url?q=https://www.google.com/url?q%3Dhttps://pubmed.ncbi.nlm.nih.gov/34245697/%26source%3Dgmail-imap%26ust%3D1685481813000000%26usg%3DAOvVaw0ExNXrScchuMD_gTYPLEFg&source=gmail-imap&ust=1694334974000000&usg=AOvVaw1a0UXXbQGWqhAhYYnR2WAo) | Holte MB, Diaconu A, Ingerslev J, Thorn JJ, Pinholt EM. | 2021 | Other topic |
| [Three-dimensional changes in the location of soft tissue landmarks following bimaxillary orthognathic surgery.](https://www.google.com/url?q=https://www.google.com/url?q%3Dhttps://pubmed.ncbi.nlm.nih.gov/33765157/%26source%3Dgmail-imap%26ust%3D1685481813000000%26usg%3DAOvVaw3HokQxpauDHC2ra6ZHPxhT&source=gmail-imap&ust=1694334974000000&usg=AOvVaw3num3PIYS29AEJH5zC5pRR) | Çoban G, Yavuz İ, Demirbaş AE. | 2021 | Other topic |
| Peri- and postoperative complications in Le Fort I osteotomies | Kotaniemi, Karoliina V. M. And Suojanen, Juho and Palotie, Tuula | 2021 | Other topic |
| Immediate dental and skeletal influence of distractor position on Surgically assisted rapid palatal expansion with or without Pterygomaxillary disjunction | Moehlhenrich, S. C. And Ernst, K. And Peters, F. And Kniha, K. And Chhatwani, S. And Prescher, A. And Danesh, G. And Hoelzle, F. And  Modabber, A. | 2021 | Not in vivo study |
| Evaluation of symmetry behavior of surgically assisted rapid maxillary expansion with simulation-driven targeted bone weakening | Chhatwani, S., Schudlich, K., Möhlhenrich, S.C., Pugachev, A., Bicsak, A., Ludwig, B., Hassfeld, S., Danesh, G., Bonitz, L. | 2021 | Other topic |
| Prevalence of malignant neoplastic oral lesions among children and adolescents: A systematic review and meta-analysis | De Paula, D.S., Nóbrega Malta, C.E., de Brito, W.H., Mota Lemos, J.V., Cetira Fillho, E.L., Gurgel Costa, F.W., Nunes Alves, A.P.N., de Barros Silva, P.G. | 2021 | SR |
| Nasopharyngeal airway and subcranial space analysis in Pfeiffer syndrome | Lu, X., Forte, A.J., Allam, O., Park, K.E., Junn, A., Alperovich, M., Steinbacher, D.M., Tonello, C., Alonso, N., Persing, J.A. | 2021 | Other topic |
| A comparison of different osteotomy techniques with and without pterygomaxillary disjunction in surgically assisted maxillary expansion utilizing modified hybrid rapid maxillary expansion device with posterior implants: A finite element study | Sankar, S.G., Prashanth, B., Rajasekhar, G., Prasad, M., Reddy, G.V., Yamini Priyanka, J.S. | 2021 | Other topic |
| Current Trends in Orthognathic Surgery in Poland-A Retrospective Analysis of 124 Cases | Zawislak, Ewa and Przywitowski, Szymon and Olejnik, Anna and Gerber,  Hanna and Golusinski, Pawel and Nowak, Rafal | 2021 | Other topic |
| New method of Le Fort I osteotomy with increased stability | Nishikubo, Shuichi and Ogisawa, Shouhei and Nakajima, Junya and Azaki,  Hiroaki and Shinozuka, Keiji and Tonogi, Morio | 2021 | Other topic |
| Effects of Surgically Assisted Rapid Palatal Expansion on Facial Soft Tissues: A Systematic Review. | Vogiatzis, F., Roussos, P., Doulis, I., Palikaraki, G., Christopoulos, P., & Sifakakis, I. | 2022 | SR |
| Three-dimensional palatal morphology and upper arch changes following nonsurgical and surgical maxillary expansion in adults | Turker, Gokhan and Coban, Gokhan and Bayraktar, Alara Unal and Kurt,  Gokmen and Kilic, Erdem and Alkan, Alper | 2022 | Other topic |
| Combined orthodontic and surgical open bite correction: Principles for Success. Part 1 | Arnett, G. William and Trevisiol, Lorenzo and Grendene, Elisabetta and  Mclaughlin, Richard P. And D'Agostino, Antonio | 2022 | Other topic |
| Combined orthodontic and surgical open bite correction: Principles for Success. Part 2 | Arnett, G. William and D'Agostino, Antonio and Grendene, Elisabetta and  Mclaughlin, Richard P. And Trevisiol, Lorenzo | 2022 | Other topic |
| Association between impaired healing after orthognathic surgery and irritable bowel syndrome: A case report and literature review | Nasrun, Nisrina Ekayani and Fujita, Keiko and Chieda, Kazumi and Abiko,  Yoshihiro and Shimo, Tsuyoshi and Akizuki, Kazuki | 2022 | Review |
| The accuracy of virtual surgical planning in segmental Le Fort I Surgery: A comparison of planned and actual outcome | Chu, Yuxian and Ye, Bin and Wu, Qionghui and Wang, Yu and Wang, Peng and  Li, Jihua | 2022 | Other topic |
| Short-Term Stability After Segmental Le Fort I Maxillary Impaction Surgery With Mandibular Autorotation in Seven High-Angle Class II  Patients: A Case Series | Takahara, Namiaki and Tomomatsu, Nobuyoshi and Hsieh, Diana and  Kurasawa, Yasuhiro and Morita, Kei-ichi and Yoda, Tetsuya | 2022 | Other topic |
| [Sequential Treatment of Extreme Maxillary Hypoplasia: A Historical Patient Report of an Edentulous Adult Patient With Ankyloglossia.](https://www.google.com/url?q=https://www.google.com/url?q%3Dhttps://pubmed.ncbi.nlm.nih.gov/34560745/%26source%3Dgmail-imap%26ust%3D1685480936000000%26usg%3DAOvVaw3wb1jXLYKKMY5BfFU6g1-K&source=gmail-imap&ust=1694334883000000&usg=AOvVaw1VR4yrOzccVjp2SyRR0Wu2) | Saint-Denis L, Simon E, Brix M. | 2022 | Other topic |
| Higher need for removal of osteosynthesis material after multi-piece Versus one-piece Le Fort I osteotomy: A retrospective study of 339 Patients | Dubron, K. And Shaheen, E. And Vaes, L. And da Costa Senior, O. And  Miclotte, I. And Politis, C. | 2022 | Other topic |
| Three-Dimensional Comparison of the Maxillary Surfaces through ICP-Type Algorithm: Accuracy Evaluation of CAD/CAM Technologies in Orthognathic Surgery | Cassoni, Andrea and Manganiello, Luigi and Barbera, Giorgio and Priore,  Paolo and Fadda, Maria Teresa and Pucci, Resi and Valentini, Valentino | 2022 | Other topic |
| Surgically assisted rapid palatal expansion: is the pterygomaxillary disjunction necessary? A finite element study | Koç, O. And Jacob, H.B. | 2022 | Other topic |
| Surgically assisted rapid palatal expansion for transverse maxillary discrepancy in adults - Case report | Jha, K. And Adhikari, M. | 2022 | Case report |
| Posterior Mandibular Segmental Split Osteotomy: A Novel Technique for Edentulous Space Reconstruction in Patients With Skeletal Class III Malocclusion | Zhu, Zihe and Ni, Shilei and Sun, Xiumei and Wu, Guomin | 2022 | Other topic |
| Accuracy of Segmented Le Fort I Osteotomy with Virtual Planning in Orthognathic Surgery Using Patient-Specific Implants: A Case Series | Rios, Olina and Lerhe, Barbara and Chamorey, Emmanuel and Savoldelli,  Charles | 2022 | Other topic |
| Effects of different distractor positions on the formation of expansion, stress and displacement patterns in surgically assisted rapid maxillary expansion without pterygomaxillary disjunction: a finite element analysis study | Koç, O. And Bolat Gumus, E. | 2023 | Other topic |
| Treatment of Severe Open Bite Malocclusion with Four-Piece Segmental Horseshoe Le Fort I Osteotomy | Hoshijima, Mitsuhiro and Oka, Naoki and Matsumura, Tatsushi and Iida, Seiji and Kamioka, Hiroshi | 2023 | Other topic |
| Alveolar bone changes after tooth-borne surgically assisted rapid Maxillary expansion: A three-dimensional study | Martin, Anais and Oyallon, Mathilde and Perrin, Jean Philippe and Durand, Thomas and Deumier, Laurent and Corre, Pierre and Renaudin, Stephane and Bertin, Helios | 2023 | Other topic |
| Three-Dimensional Accuracy and Stability of Personalized Implants in Orthognathic Surgery: A Systematic Review and a Meta-Analysis | Diaconu, Alexandru and Holte, Michael Boelstoft and Berg-Beckhoff,  Gabriele and Pinholt, Else Marie | 2023 | Other topic |
| Effects of anterior maxillary distraction compared to lefort-1 osteotomy And total maxillary distraction osteogenesis for treating hypoplastic Maxilla in patients with cleft lip and palate- A systematic review and Meta-analysis | Kaur, Harneet and Grover, Seema and Singaraju, Gowri Sankar and Sidhu, M. S. And Jaglan, Archana and Dogra, Namrata | 2023 | Other topic |

Legend

RCT= randomized clinical trial; SR= Systematic Review
